# Technology-Assisted Home Care for People With Dementia and Their Relatives: Scoping Review

**DOI:** 10.2196/25307

**Published:** 2021-01-20

**Authors:** Sarah Palmdorf, Anna Lea Stark, Stephan Nadolny, Gerrit Eliaß, Christoph Karlheim, Stefan H Kreisel, Tristan Gruschka, Eva Trompetter, Christoph Dockweiler

**Affiliations:** 1 Institute for Educational and Health-care Research in the Health Sector Bielefeld University of Applied Sciences Bielefeld Germany; 2 Centre for ePublic Health Research School of Public Health Bielefeld University Bielefeld Germany; 3 Institute for History and Ethics of Medicine Interdisciplinary Center for Health Sciences Martin Luther University Halle-Wittenberg Halle Germany; 4 Nursing Science Staff Unit Franziskus-Hospital Harderberg Niels-Stensen-Kliniken Georgsmarienhütte Germany; 5 Innovation & Research, Executive Department Evangelisches Klinikum Bethel University Hospital OWL – Campus Bielefeld-Bethel Bielefeld Germany; 6 Division of Geriatric Psychiatry, Department of Psychiatry and Psychotherapy Evangelisches Klinikum Bethel University Hospital OWL – Campus Bielefeld-Bethel Bielefeld Germany; 7 Faculty of Social Studies Bielefeld University of Applied Sciences Bielefeld Germany

**Keywords:** dementia, home care, assistive technologies, scoping review

## Abstract

**Background:**

Assistive technologies for people with dementia and their relatives have the potential to ensure, improve, and facilitate home care and thereby enhance the health of the people caring or being cared for. The number and diversity of technologies and research have continuously increased over the past few decades. As a result, the research field has become complex.

**Objective:**

The goal of this scoping review was to provide an overview of the research on technology-assisted home care for people with dementia and their relatives in order to guide further research and technology development.

**Methods:**

A scoping review was conducted following a published framework and by searching 4 databases (MEDLINE, CINAHL, PsycInfo, and CENTRAL) for studies published between 2013 and 2018. We included qualitative and quantitative studies in English or German focusing on technologies that support people with dementia or their informal carers in the home care setting. Studies that targeted exclusively people with mild cognitive impairment, delirium, or health professionals were excluded as well as studies that solely consisted of assessments without implication for the people with dementia or their relatives and prototype developments. We mapped the research field regarding study design, study aim, setting, sample size, technology type, and technology aim, and we report relative and absolute frequencies.

**Results:**

From an initial 5328 records, we included 175 studies. We identified a variety of technology types including computers, telephones, smartphones, televisions, gaming consoles, monitoring devices, ambient assisted living, and robots. Assistive technologies were most commonly used by people with dementia (77/175, 44.0%), followed by relatives (68/175, 38.9%), and both target groups (30/175, 17.1%). Their most frequent goals were to enable or improve care, provide therapy, or positively influence symptoms of people with dementia (eg, disorientation). The greatest proportions of studies were case studies and case series (72/175, 41.1%) and randomized controlled trials (44/175, 25.1%). The majority of studies reported small sample sizes of between 1 and 50 participants (122/175, 69.7%). Furthermore, most of the studies analyzed the effectiveness (85/233, 36.5%) of the technology, while others targeted feasibility or usability or were explorative.

**Conclusions:**

This review demonstrated the variety of technologies that support people with dementia and their relatives in the home care setting. Whereas this diversity provides the opportunity for needs-oriented technical solutions that fit individual care arrangements, it complicates the choice of the right technology. Therefore, research on the users’ informational needs is required. Moreover, there is a need for larger studies on the technologies’ effectiveness that could contribute to a higher acceptance and thus to a transition of technologies from research into the daily lives of people with dementia and their relatives.

## Introduction

About 50 million people worldwide suffer from dementia, and there are almost 10 million new cases every year [1]. Dementia is an umbrella term that describes a syndrome, usually of a chronic nature, in which there is a disorder of several higher cortical functions: memory, thinking, orientation, language, judgment, and learning [2]. Due to the disease, people with dementia are restricted in their activities of daily life. Furthermore, the prevalence of challenging behaviors such as anxiety, hallucinations, delusions, or disinhibition is high [3-5]. Over the course of the disease, different needs for support occur. These needs range from assistance with activities of daily living (eg, personal hygiene), psychosocial support (eg, coping with the disease), and help with disorientation [6]. Nevertheless, people with dementia want to live at home as long as possible [7,8], and moving to a new environment (eg, long-term care) increases confusion, disorientation, and behavioral symptoms [9,10]. Home care is mostly provided by relatives, which can result in conflicts between the support needs and requirements of those affected and the available resources of the informal caregivers. Relatives often feel obliged [11] and have a high burden of care [12,13]. This causes tension in the family system and a feeling of being overwhelmed. As a result, the quality of care cannot be maintained, and even a move to a long-term care setting is necessary [14].

Assistive technologies can potentially maintain and support home care arrangements and consequently avoid or postpone residential care [15,16]. They have various aims, such as supporting communication [17-19], providing timely education or therapy for people with dementia and their relatives [20,21], offering assistance with daily activities (eg, cooking) [22], or reducing disease-related risks (eg, getting lost) [23]. Thereby, they encourage independence and social inclusion [15,16,24]. On the other hand, a recent study did not demonstrate a significant reduction in caregiver burden, anxiety, and depression in a large study population [25]. The evidence therefore does not seem to be clear. Barriers to the use of assistive technologies included perceptions of the high cost of formal assistive technologies; dilemmas regarding the timing and stage of technology use; and a lack of information and support from formal health and social care services about access, sources, timing, and options for use [26].

With regard to the different support domains, there is a wide diversity of assistive technologies, ranging from simple applications to complex multicomponent technologies. Assistive technologies can be defined as technological devices aimed “(…) to maintain or improve an individual's functioning and independence to facilitate participation and to enhance overall well-being” [27]. Research and development in this field has increased significantly in recent years due to technological progress, increasing demand and research funding [28]. However, the research area is very confusing due to the large number of different technologies with varying degrees of development for different target groups as well as various objectives of these technologies. We therefore conducted a scoping review to provide an overview of existing research on assistive technologies for people with dementia and their families in the home setting, guided by the research question: What types of assistive technologies are described in the current scientific literature for people with dementia and family carers to support care in the home setting?

## Methods

We conducted a scoping review following the steps described by Arksey and O’Malley [29] with an extension by Levac et al [30]. The steps include (1) formulating the research question; (2) identifying relevant studies; (3) selecting relevant studies; (4) charting the data; (5) collating, summarizing, and reporting results; and (6) consultation. We did not publish a protocol for this review and used PRISMA-ScR (Preferred Reporting Items for Systematic Reviews and Meta-Analyses extension for Scoping Reviews) for reporting of this review [31].

### Eligibility Criteria

We included publications with qualitative or quantitative study designs focusing on technologies supporting people with dementia or their informal caregivers in the home care setting published between January 2013 and October 2018 in the German or English language. The time restriction of 5 years prior to the search date was chosen due to the rapid and significant changes that are made in the digital sector. We included studies conducted in day care centers and nursing homes because some of the technologies tested in these settings are also described as suitable for use in the home setting.

We excluded studies targeting people with mild cognitive impairment or delirium only as well as studies on electronic aids (eg, electric wheelchair) or technologies for the sole purpose of dementia assessment or diagnostics without any implication for the home care of people with dementia. Additionally, we excluded studies on technologies that are exclusively used by health professionals. We also excluded studies that only reported on technical aspects or parts of a technology (eg, interfaces or prototypes) as well as systematic reviews and study protocols.

### Search Process

We searched the databases MEDLINE, CINAHL, PsycInfo, and CENTRAL up to October 2018. To develop the search strategy, the review team brainstormed potentially important search terms, scoped relevant studies for controlled vocabulary, and searched the MeSH browser for relevant MeSH terms mapped to uncontrolled vocabulary. The search strategy was reviewed internally via the Peer Review of Electronic Search Strategies (PRESS) guideline [32]. Two review authors (AS, SP) independently screened titles, abstracts, and full texts for inclusion. In cases of uncertainty, a third author (SN) was consulted.

### Data Extraction and Critical Appraisal

Two study authors (AS, SP) extracted the following study characteristics using a standardized data extraction sheet and resolving differences by discussion: authors, year of publication, study design, study aim, country, setting, sample size, name of technology, type of technology, and aim of technology.

We did not perform a standardized critical appraisal of the included studies with, for example, the Cochrane Risk of Bias tool, since our goals were to give an overview and map out topics.

### Synthesis

We report the results in a structured and narrative synthesis, graphically, and in tabular form. Therefore, we grouped the studies’ technologies thematically and mapped out the study designs, technology groups, and goals of the studies. Additionally, we compared the settings, target groups, and sample sizes. The trends in publication numbers as well as the inclusion of the target groups were analyzed. We report the results with descriptive statistics in absolute and relative frequencies. A brief report on the nonformalized consultation process by means of expert discussions at 2 conferences is incorporated in the discussion.

## Results

**Figure 1:**
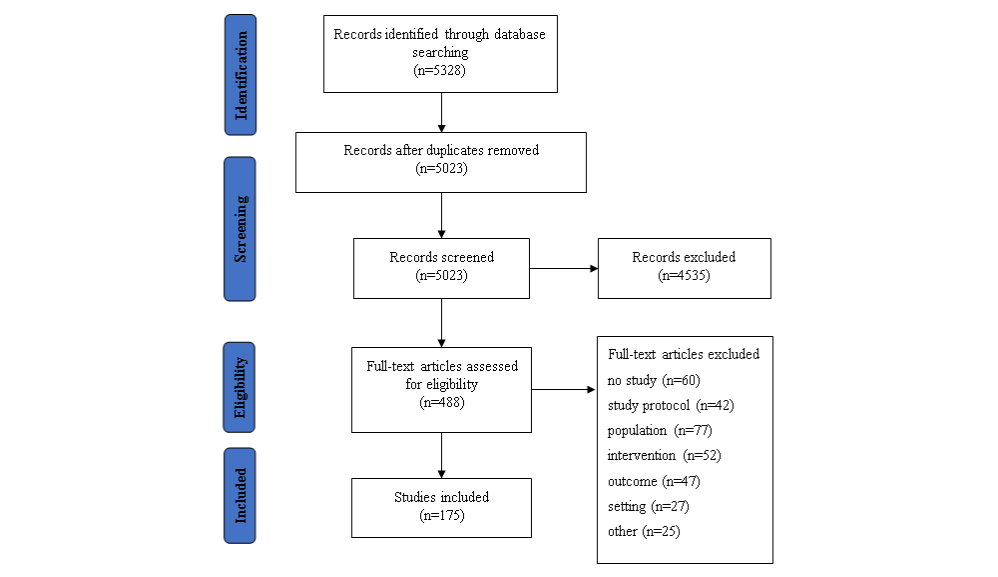
PRISMA flowchart.

The database search identified 5328 titles. After abstract and full-text screening, 158 publications describing 175 studies with a total of 10,167 participants were included. See the PRISMA flowchart [33] for the illustration of the search process (Figure 1) and the multimedia appendices for the studies’ references (Multimedia Appendix 1) and study details (Multimedia Appendices 2-4). The divergent number of studies and articles can be explained by the fact that several different case studies on different technologies are combined in 1 article. These studies do not meet the criteria of case series or multiple case studies. In addition, different studies, which varied in design, were described in 1 article.

**Figure 2:**
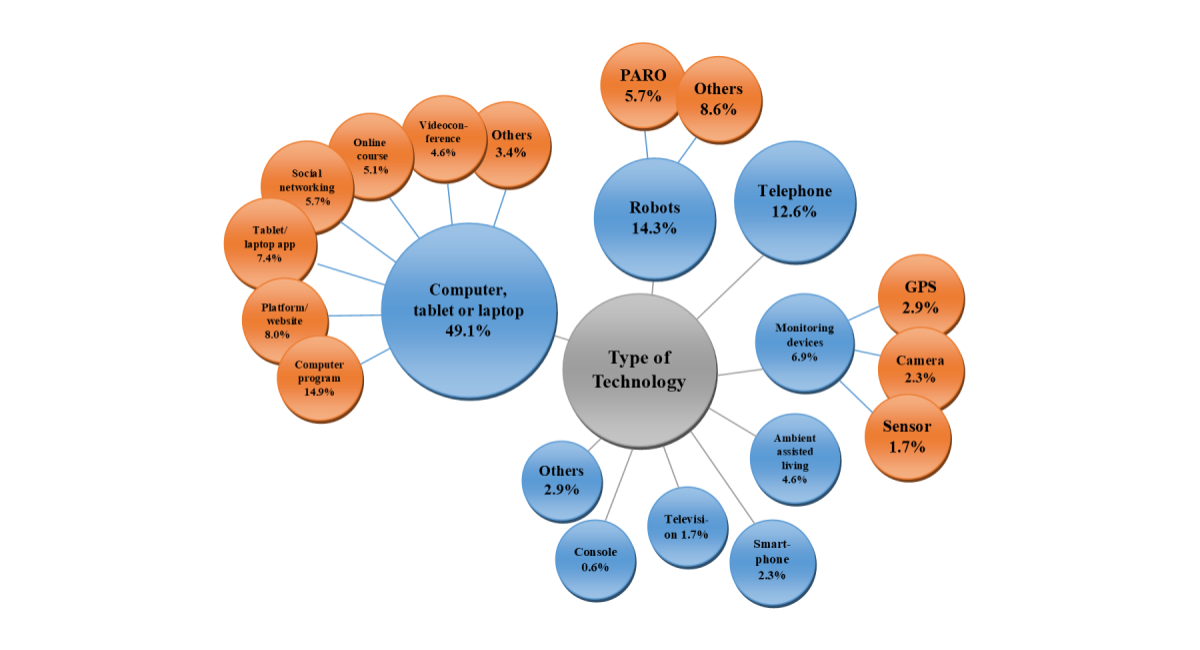
Relative frequencies of the types of technologies in the included studies (n=175).

In order to answer the question of existing assistive technologies to support people with dementia and their relatives, a diagram was created clustering the different types of technologies under investigation (Figure 2).

About half of the studies (86/175, 49.1%) addressed different applications on computers, laptops, or tablets. Furthermore, robots (25/175, 14.3%) and telephone interventions (22/175, 12.6%) were frequently studied. Among robotic systems, PARO (PARO Robots US Inc, Itasca, IL) was the most commonly covered technology (10/175, 5.7%). Other technologies such as gaming consoles (1/175, 0.6%), apps on smartphones (4/175, 2.3%), ambient assisted living (8/175, 4.6%), and monitoring systems (12/175, 6.9%) were covered less frequently.

The studies can be classified according to different study characteristics. Focusing on the target group, technologies were primarily used by people with dementia (77/175, 44.0%), their relatives (68/175, 38.9%), or both target groups (30/175, 17.1%). With regard to the technology groups presented, most of the studies in which robots were tested were conducted with people with dementia (23/25, 92%). Computer programs (16/20, 80%) and apps on tablets (9/11, 82%) were also tested most commonly with people with dementia. Telephone-based interventions (22/22, 100%), internet courses (9/9, 100%), special websites (9/9, 100%), and online social networking or support groups (8/10, 80%) were almost exclusively related to family carers. When both target groups were addressed, monitoring (7/30, 23%) and ambient assisted living systems (5/30, 17%) were examined more frequently.

With respect to the setting, 60.0% (105/175) of the studies were conducted at home, 20.0% (35/175) in nursing homes, 11.4% (20/175) in day care centers, and 5.1% (9/175) in more than one setting. Concerning the technology groups, more than two-thirds of the studies with robots were conducted in nursing homes (17/25, 68%). Telephone interventions (22/22, 100%), apps on computers (6/6, 100%), and monitoring systems (9/12, 75%) were tested exclusively or predominantly in the home setting. Computer programs were tested more frequently in day care centers (8/18, 44%) and nursing homes (5/18, 28%) than at home (3/18, 17%). Furthermore, almost all studies that focused on the relatives took place in the home environment (66/68, 97%), and studies focusing on both target groups were more likely to take place at home (19/30, 63%) than in nursing homes (8/30, 27%). People with dementia were most often studied in nursing homes (27/77, 35%). However, a similar proportion of this target group was assessed at home (20/77, 26%) and in day care centers (19/77, 25%).

Overall, the number of included publications per year was relatively stable over time, with a mean of 26 publications per year. The number varies between a minimum of 21 publications in 2016 and a maximum of 34 publications in 2017. Table 1 shows the number of publications per year and target group of the technical intervention.

**Table 1:** Absolute number of publications by publication year (n=158).

Target group	Publication year					
	2013	2014	2015	2016	2017	2018
People with dementia	10	10	10	5	17	11
Caregivers	7	12	15	10	13	10
Both people with dementia and caregivers	7	5	4	6	4	2

There is a noticeable increase in the number of publications focusing on people with dementia between 2016 (n=5) and 2017 (n=17). Additionally, it becomes clear that the number of publications with the target group of relatives increases significantly from 2013 to 2015, and they represent the largest target group from 2014 to 2016. From 2017 onwards, this trend changed, and most interventions investigated assistive technologies for people with dementia. In 2018, the number of publications of these target groups is approximately the same (caregiver n=10; people with dementia n=11). Few of the studies focused on both target groups as users of technologies.

Regarding the number of participants, the majority of studies included 1 to 50 persons (1-10: 59/175, 33.7%; 11-50: 63/175, 36.0%). Table 2 shows the number of publications by the technologies’ target group and sample size. In smaller studies with a maximum of 10 participants, the proportion of publications about people with dementia (38/59, 64%) was particularly high. In studies with 51 or more participants, the majority of studies focused on caregivers (51-100: 12/19, 63%; 101-200: 11/14, 79%; ≥201: 9/16, 56%).

**Table 2:** Absolute number of publications by number of participants (n=175).

Target group	Number of categorized participants				
	1-10	11-50	51-100	101-200	≥201
People with dementia	38	26	5	3	5
Caregivers	10	24	12	11	9
Both people with dementia and caregivers	11	13	2	0	2

The assistive technologies were also investigated according to their study designs. Case studies represented 33.1% (58/175), 25.1% (44/175) were randomized controlled trials (RCTs), and 10.3% (18/175) were pre-post studies. Only a small proportion of the studies used case series (14/175, 8.0%), an exclusively qualitative design (14/175, 8.0%), a controlled trial (7/175, 4.0%), or a cross-sectional design (7/175, 4.0%). The remaining studies were classified as having “other” designs (13/175, 7.4%).

Grouping study designs by target groups, the largest percentage of studies focusing on patients with dementia used case studies (37/77, 48%). Subsequently, case series and RCTs represented the second largest proportion for this target group (11/77 each, 14%). Regarding the relatives, most studies used RCTs (30/68, 44%) and pre-post designs (14/68, 20.5%). When both target groups were investigated, case studies were mostly utilized (14/30, 47%). Case studies in general mainly consisted of people with dementia (37/58, 64%) or both target groups (14/58, 24%). In the qualitative studies, all target groups were examined with similar frequency (people with dementia and both groups: 5/14, 36%; relatives: 4/14, 29%).

When stratified by setting, in day care centers, mainly case studies and series were conducted (18/20, 90%). Case studies also accounted for half of the research in nursing homes (18/35, 51%). In contrast, the number of RCTs was highest in the home setting (35/105, 33.3%), followed by case studies and case series (29/105, 27.6%) as well as pre-post studies (15/105, 14.3%). In addition, most qualitative studies were conducted at home (10/14, 71%). Regarding the number of participants and study designs, 25% (11/44) of RCTs incorporated 11-50 people, and 30% (13/44) of RCTs incorporated each of 51-100 and ≥201 persons. With respect to the technology group, most of the RCTs and controlled trials were performed with testing telephone interventions (18/51, 35%), robots (8/51, 16%), and internet courses (7/51, 14%). In the case studies and case series, applications on computers, tablets, and laptops (38/72, 53%) as well as robots (10/72, 14%) were examined most frequently.

The nature of assistive technologies is particularly determined by its purpose. For better comparability, 8 categories of technology aims were formed. As some technologies had multiple functions, they were assigned to more than 1 category in order not to simplify their complexity.

The largest proportion of technologies aimed to enable or support therapeutic or caring interventions (85/308, 27.6%). Therapeutic technology–supported interventions included online therapy for people with dementia or their caregivers [34-36] or art therapeutic interventions via a technical device (n=31) [37,38]. Care interventions sought to increase the safety of people with dementia, for example by detecting the danger of falling at an early stage (n=54). A specific example was the study by Bayen et al [39], which analyzed how continuous video monitoring and review of falls of individuals with dementia can support better quality of care. Abbate et al [40] used a wireless accelerometer and electroencephalograph logger integrated in a minimally invasive monitoring sensor system with the aim of detecting possible falls and their causes. Care interventions also included online training programs for relatives with the goal of improving caring by trying to facilitate everyday life (eg, dealing with people with dementia). The European project STAR offers caregivers of people with dementia (both formal and informal) online training in order to better understand the disease and provide higher quality care [41]. Furthermore, 25.3% (78/308) of the technologies aimed to positively influence the symptoms of people with dementia such as disorientation or fear. Other technologies have been used to increase the knowledge of people with dementia or their relatives, such as through special websites (34/308, 11.0%), to enable or improve communication (29/308, 9.4%; eg, by providing an easy-to-use interface that allows people with dementia to contact their relatives) [42], or to enhance the skills of people with dementia in particular (20/308, 6.5%). Skill improvement included abilities such as remembering, orientation, and movement. This involved games that increased cognitive performance [43] or interventions to improve mobility [44]. An equal share of technologies (12/308 each, 3.9%) wanted to support activities of daily life (eg, by guiding people with dementia in their activities [22,45]) or improve engagement (eg through entertaining games [46]). “Other aims” were described for 12.3% (38/308) of the technologies. Overall, the objectives of the technologies were very broad. Due to the high complexity of technologies and the poor reporting, categorization of technology aims can only be based on the information provided by the studies. Therefore, the categories cannot be clearly distinguished from each other. In this context, caring tends to be a superficial main category, as many authors merely state an improvement in care provision as an aim, without describing in detail what the intervention specifically addressed in terms of needs.

A large percentage of the studies aimed to investigate the effects of the technology, either in terms of demonstrating effectiveness (85/233, 36.5%) or, more generally, by evaluating the assistive technologies (23/233, 9.9%). With regard to factors influencing intervention effects, few of the studies had the goal of measuring acceptance (16/233, 6.9%) or usability (23/233, 9.9%). In order to gain a first or deeper insight into the possible modes of action of the technologies, the minority were labelled as exploratory (22/233, 9.4%) or feasibility studies (31/233, 13.3%).

The objective of analyzing effects was similarly high in all studies regardless of the target group (people with dementia: 42/100, 42.0%; relatives: 33/84, 39%). The effectiveness was tested especially in studies in day care centers (17/26, 65%). Furthermore, many of the case studies pursued this aim (27/80, 34%). Case studies often examined the feasibility (13/80, 16%) or usability (11/80, 14%), or they were used for exploration (8/80, 10%). Feasibility, in turn, was given as the aim of the study in both target groups equally frequently (people with dementia: 13/100, 13%; relatives: 11/84, 13%). Many of these feasibility studies investigated technical interventions on computers, tablets, or laptops (21/31, 68%).

## Discussion

Overall, this scoping review gives a comprehensive overview of the current literature and shows the diversity of assistive technologies for people with dementia and their family caregivers. There is a comparable amount of studies focusing on people with dementia as well as their caregivers. On the one hand, this demonstrates the increased availability of assistive technologies for informal caregivers; on the other hand, this demonstrates recognition of family members and people with dementia as consumers.

Many of the studies had the aim of demonstrating the effectiveness of the technology, although most of them were case studies with small sample sizes. This indicates that many of the technologies were rather rudimentarily tested, and only a very limited number of findings about effects or feasibility has been established, resulting in low confidence in the results. However, this seems odd, as usually a lot of financial and personal resources have to be invested in the development of a technology. Consequently, it would be reasonable to test them adequately. However, we acknowledge that it is difficult, especially for profit-oriented companies, to scientifically test the effectiveness of their developed technologies due to potential conflicts of interest. Evidence for the effectiveness of interventions through RCTs and controlled trials is more prevalent, although still limited, for telephones, robots, and internet courses. In total, a large proportion of studies was aimed at the technical evaluation, exploration, usability, or feasibility of an assistive technology. This indicates that many technologies for people with dementia and their informal carers are still in an early development stage. There is a need for larger studies of technologies’ effectiveness. A broad evidence base about the benefits and risks of technologies for users is crucial to promote their acceptance and therefore achieve a transition of technologies from research into the daily lives of people with dementia and their relatives [47]. Successful technology arrangements were often characterized by pragmatic adaptation and combination of new with old equipment by the people with dementia or their caregivers [48,49].

We found heterogeneous technologies in our review. Telephone interventions have been frequently analyzed. A major advantage of telephone interventions is that there is no need to purchase expensive technologies because existing resources can be used. Furthermore, the technology is already known, used, and therefore accepted by the users. This could have the advantage, especially for people with dementia, that they could still use this technology in a later phase of the illness without being challenged with learning something new. Hence, use in everyday life seems more easily compared to other technologies. Internet courses are low-threshold interventions that can provide timely education for caregivers and reduce stress [50]. Additionally, they are relatively low-cost developments compared to, for example, a robotic system. Robots, by contrast, are complex technologies that can provide support in many ways (eg, socioemotional support, taking over household tasks, guiding actions, or recognizing and intervening in changing or dangerous situations). As we stated before, one robotic system, called PARO, has been of great interest for researchers. Studies using PARO were mostly placed in nursing homes or day care centers and evaluated its effectiveness. Reviews, which specifically analyzed robots for older people with and without dementia, found positive but not always significant effects on behavioral and emotional aspects, quality of life, and communication [51-53].

In a large number of interventions, both target groups were involved (eg, in order to individualize the interaction between the technology and people with dementia, their family chose photos, music, or videos [38]). Few of the technologies were designed to involve both target groups with the aim of supporting their interaction or communication [54]. This again shows the variety of application areas regarding assistive technologies for people with dementia and their family caregivers. To ensure that results are generalizable, we suggest that future reviews analyzing the effectiveness of assistive technologies focus on a group of technologies that are similar regarding their technical components, aims, and target groups.

Corresponding to the last step of the scoping review process model, the results of the scoping review were presented and discussed at 2 conferences in the form of a poster presentation and a lecture by experts in the field of health care research and practical care of people with dementia [55,56]. The main questions asked referred to the acceptance and adoption of the technologies in the household. The topic of acceptance of the technologies is hardly represented in the studies. Studies referring to the fact that the users have accepted the technologies and integrated them into their households usually provided a detailed description of how this process took place and whether there were any facilitation efforts (eg, external support by the project team) or how the acceptance was determined. Studies that explicitly investigated acceptance measured the use of the technology, user attitude, user mood (eg, relaxed or joyful), or user satisfaction [37,57,58]. Cristancho-Lacroix et al [59] reported a lack of acceptance, which was measured using qualitative data. It remains unclear which specific aspects have a negative impact on acceptance. Few of the studies explicitly reported on challenges in using the technology or barriers to use [37]. Especially with the large number of case studies, we would have expected more detailed information regarding this issue. In addition, this information could be of importance in determining whether interventions can be recommended by health care professionals or so that people with dementia and their families can decide whether to use a technology. Based on the experts’ comments, we conclude that more and in-depth evidence is needed about the user acceptance of such technologies. Studies should be based on relevant theories such as the unified theory of acceptance and use of technology (UTAUT) [60], in order to gain meaningful and valid results with regard to the implementation. Specific concepts like the non-adoption, abandonment, scale-up, spread, sustainability (NASSS) framework can be helpful to evaluate factors influencing the adoption of technologies in order to plan an effective implementation [61]. It also requires industry and service providers to take a user-centric approach to design and deployment [62]. People with dementia and their caregivers identified clear information pathways for assistive technologies as essential for both service providers and service commissioners [63].

Due to the exploratory nature of the scoping review, it has to be considered that studies may have been overlooked despite the broad search because of the restrictions in databases, languages, and period of time. Because of the broad research question and heterogeneous study situation, a more in-depth analysis of specific technologies was not suitable. Furthermore, studies whose results did not demonstrate acceptance or positive outcomes may not have been published (publication bias). A particular difficulty arose in extracting data from studies and classifying technologies due to the poor reporting of the studies. This was especially prevalent for the methodological approach of the studies, description of the users, and use of the assistive technologies. In contrast, these studies focused more on technical aspects of the technologies, such as the design of an interface or data streams of systems. We still included studies with a focus on technical aspects when they reported how the technology was tested, because that was of particular interest in our review. In these cases, it was also more difficult to determine the purpose of the technology. Therefore, these were categorized based on the authors' explanations. A standardized description of the technologies using the CONSORT EHEALTH [64] or the TIDieR [65] checklist could contribute to a better understanding. In addition, the user group of people with dementia was insufficiently described in some cases. This refers to the existence of a concrete diagnosis of dementia and its testing, form of dementia, and symptoms of the disease, especially with regard to communication skills. Some participants were described as having dementia, but in the testing of cognitive abilities, they only showed limitations in the area of mild cognitive impairment. This makes it difficult to identify relevant studies and assess the transferability of study results.

Overall, there is great diversity in assistive technologies for people with dementia and their family caregivers. This becomes particularly clear when analyzing the different types of technologies and their purposes. One advantage of this diversity is that different technologies can address different problems and needs. Thus, the repertoire for the solution of these different problems is extended by technical interventions. This gives people with dementia, their relatives, and health care professionals more options for tailoring care arrangements to their needs. On the other hand, the diversity of technologies makes it more difficult for end users in particular to gain an overview of existing possibilities. This is especially true when technologies are developed for a broad group of users (eg, elderly people or people with cognitive disabilities). Here, it is even more complicated to decide on the appropriateness of the application of a specific assistive technology in a specific case. This results in the necessity of a user-oriented database to inform potential users about the available technologies. We recommend that the database includes various information of the technology, such as specific target group, aims, effectiveness, and user experiences. Therefore, an analysis of the users’ informational needs would be beneficial. Furthermore, there is a major need for well-developed and tested interventions. This includes the measurement of not only (health) care outcomes but also feasibility and acceptability. Participatory design and development processes have to be implemented to fulfill the needs as well as acceptability, usability, and ethical issues of future users [23,66,67]. It is possible that case studies have remained at this level of research with no apparent follow-up projects because only low acceptance or effects have been identified. At the same time, there is a broad need for (1) technologies to assist people with dementia in several areas, (2) identification of the characteristics these technologies should have based on the users’ needs, and (3) information on these technologies that is required by the users [68]. We believe that this scoping review can contribute to further guide research on assistive technologies for people with dementia and their family caregivers.

## Appendix

Multimedia Appendix 1 Search strategy for MEDLINE.

Multimedia Appendix 2 Study details—people with dementia.

Multimedia Appendix 3 Study details—informal carers.

Multimedia Appendix 4 Study details—both target groups.

## Abbreviations

NASSS: non-adoption, abandonment, scale-up, spread, sustainability
PRESS: Peer Review of Electronic Search Strategies
PRISMA-ScR: Preferred Reporting Items for Systematic Reviews and Meta-Analyses extension for Scoping Reviews
RCT: randomized controlled trial
UTAUT: Unified theory of acceptance and use of technology
