# Supplementary material for: Technology-Assisted Home Care for People With Dementia and Their Relatives: Scoping Review
Source: JMIR Aging. 2021 Jan 20;4(1):e25307. doi: 10.2196/25307 (PMC7857954; doi:10.2196/25307)
Supplement: Multimedia Appendix 1 [file aging_v4i1e25307_app1.docx]

Search strategy for MEDLINE

(dementia[mesh] OR dement*[TIAB] OR alzheimer* OR lewy OR (creutzfeldt OR jcd[TIAB] OR cjd[TIAB]) OR huntington OR binswanger*) AND (Technology[mesh] OR Internet[mesh] OR wearable electronic devices[mesh] OR video recording[mesh] OR video games[mesh] OR reminder systems[mesh] OR mobile applications[mesh] OR user-computer interface[mesh] OR geographic information systems[mesh] OR self-help devices[mesh] OR robotics[mesh] OR monitoring, ambulatory[mesh] OR signal processing, computer-assisted[mesh] OR therapy, computer-assisted[mesh] OR facial recognition[mesh] OR Computer-Assisted Instruction[mesh] OR Telecommunications[mesh] OR audiovisual aids[mesh]) NOT (Review[PT] OR Meta-Analysis[PT])
