# Supplementary material for: Technology-Assisted Home Care for People With Dementia and Their Relatives: Scoping Review
Source: JMIR Aging. 2021 Jan 20;4(1):e25307. doi: 10.2196/25307 (PMC7857954; doi:10.2196/25307)
Supplement: Multimedia Appendix 2 [file aging_v4i1e25307_app2.docx]

**Appendix 2: Study Details – People with Dementia**

| **Author** | **Study Design** | **Study aim** | **Country**  **Setting** | **Number of participants** | **Technology name** | **Technology group** | **Technology aim** |
| --- | --- | --- | --- | --- | --- | --- | --- |
| Abbate et al. 2014 [1] | Case study | Usability and acceptability | Canada  nursing home | 4 | MIMS | Monitoring sensor | Improvement of care and safety (preventing/fast reaction to falls) |
| Astell et al. 2016 [2] | RCT | Usability and evaluation the concept of familiarity in gameplay | UK  care service room | 30 | Solitaire and Bubble Xplode | Tablet  app  gaming | Enabling recreation |
| Astell et al. 2018 [3] | Pre-post study | Effectiveness | UK  day care and nursing home | 98 | Computer Interactive Reminiscence and Conversation Aid (CIRCA und  CIRCA-WB) | Computer program | Improvement of communication and enabling reminiscence therapy |
|  | Repeated measures study |  |  | 53 |  |  |  |
| Bayen et al. 2017 [4] | Case series | Acceptability and effectiveness | USA  nursing home | 38 | Unclear | Monitoring  video  app | Improvement of care (prevention and reduction of falls) |
| Begum et al. 2013 [5] | Case series | Feasibility and  usability | Canada  home (lab) | 10 | Ed | Robot | Improvement of performing adls |
| Begum et al. 2015 [6] | Case series | Feasibility | Canada  home (lab) | 20 | Ed | Robot | Improvement of performing adls |
| Bejan et al. 2017 [7] | Case series | Usability and exploration | Germany  nursing home | 14 | Microsoft PixelSense  app unclear | Surface table computer  app | Improvement of symptoms and communication, enabling reminiscence therapy |
|  |  | Evaluation and  acceptability |  | 5 |  |  |  |
| Bejan et al. 2018 [8] | Case series | Evaluation and  acceptability | Germany  nursing home | 16 | Unclear | Surface table computer and tablet app | Improving engagement, enabling recreation and reminiscence therapy |
|  |  |  |  | 5 |  | Surface table computer and tablet app |  |
|  |  |  |  | 12 |  | TV and tablet  movie |  |
| Bier et al. 2018 [9] | Case study | Exploration | Canada  home | 1 | Various | Smartphone  app | Various |
| Boyd et al. 2017 [10] | Qualitative study | Feasibility | UK  home | 24 | Unclear | Tablet  app | Improvement of performing adls |
| Burdea et al. 2015a [11] | Pre-post design | Evaluation, feasibility and acceptability | USA  nursing home | 10 | the BrightBrainer | Computer  gaming  virtual reality | Improvement of symptoms  and skills |
| Burdea et al. 2015b [12] | Case study | Evaluation and  system evaluation | USA  unclear | 1 | the BrightBrainer | Computer  gaming  virtual reality | Improvement of symptoms  and skills |
| Cheong et al. 2015 [13] | CT | Effectiveness | South Korea  public health care center | 427 | Unclear | Telemedicine | Improving access to health care |
| Chu et al. 2017 [14] | Cross sectional study | Exploration and evaluation | Australia  nursing home | 139 | Sophie and Jack | Robot | Improvement of engagement, communication and care |
| Cutler et al. 2016 [15] | Case series | Evaluation and exploration | UK  unclear | 29 PwD plus caregivers (n unclear) | various e.g. Nintendo Wii Mario Kart, Nintendo DS card games,  Apple iPad YouTube | Tablet and gaming console various | Improvement of engagement |
| Dethlefs et al. 2017 [16] | Case series | Feasibility | UK  unclear | 23 (10 PwD) | Wizard-of-Oz interface | Computer  gaming | Improvement of skills |
| Ehret et al. 2017 [17] | Case series | Development, acceptability and evaluation | Germany  day care center | 14 (12 PwD) | Unclear | Tablet  gaming | Improvement of skills (memory) |
| Evans et al. 2016 [18] | Case study | Evaluation and exploration | UK  nursing home and home | 48 | Unclear | Personal message card | Enabling presence therapy |
| Foloppe et al. 2018 [19] | Case study | Feasibility | France  home | 2 | Unclear | Laptop  gaming  virtual reality | Improvement of performing adls (cooking) |
| Gustafsson et al. 2015 [20] | Case study | Exploration | Sweden  nursing Home | 4 | JustoCat | Robot | Enabling reminiscence therapy and improvement of symptoms and communication |
|  | Qualitative study | Usability, functionality and evaluation |  | 14 (3 caregiver) |  |  |  |
| Imbeault et al. 2018 [21] | Case study | Feasibility | Canada  home | 2 | Unclear | Tablet  app | Improvement of engagement |
| Jentoft et al. 2014 [22] | Qualitative study | Exploration | Norway  home | 16 | Unclear | Remote control | Improvement of performing adls (watching TV) |
| Jones et al. 2018 [23] | Secondary data analysis (RCT) | Effectiveness | Australia  nursing home | 138 | PARO | Robot | Enabling reminiscence therapy and improvement of symptoms  and communication |
| Kerssens et al. 2014 [24] | Repeated measures study | Feasibility | USA  assisted living and nursing home | 14 | the Companion | Touchscreen computer  program | Enabling reminiscence therapy and improvement of symptoms |
|  |  |  | USA  nursing home | 21 |  |  |  |
| Kerssens et al. 2015 [25] | Case study | Feasibility and usability | USA  home | 14 | the Companion | Touchscreen computer  program | Enabling reminiscence therapy and improvement of symptoms |
| Khosla et al. 2017 [26] | Longitudinal study | Effectiveness and acceptability | Australia  nursing home | 115 | Matilda | Robot | Improvement of engagement |
| Kouroupetroglou et al. 2017 [27] | Case study | Study 1: exploration | Ireland  nursing home and home | 5 | MARIO | Robot | Improvement of communication |
|  |  | Study 2 : exclusion setting hospital |  |  |  |  |  |
| Kuwamura et al. 2016 [28] | Case study | Exploration | Japan  group home | 3 | Telenoid | Robot | Initiation of communication |
| Lancioni et al. 2013a [29] | Case study | Effectiveness and social validation | Italy  day care center | 5 | Unclear | Orientation system  sound and light | Improvement of skills (orientation) |
| Lancioni et al. 2013b [30] | Case study | Effectiveness | Italy  day care center | 3 | Pinnacle Studio software | Computer  program | Improvement of performing adls |
|  |  |  |  | 4 | Unclear | Orientation system  sound and light | Improvement of skills (orientation) |
| Lancioni et al. 2014a [31] | Case study | Effectiveness | Italy  day care center | 3 | Pinnacle Studio software | Computer  program | Improvement of performing adls |
|  |  |  |  | 3 | Unclear | Orientation system  sound and light | Improvement of skills (orientation) |
| Lancioni et al. 2014b [32] | Case study | Effectiveness | Italy  day care center | 4 | Pinnacle Studio software | Computer  program | Improvement of performing adls |
|  |  |  |  | 4 | Unclear | Computer  microswitch  program | Enabling recreation |
| Lancioni et al. 2015 [33] | Case study | Effectiveness | Italy  day care center | 3 | Unclear | Computer  microswitch  program | Enabling recreation |
|  |  |  |  | 3 | Unclear | Computer  microswitch  program | Improvement of skills (mobility) |
|  |  |  |  | 3 | Unclear | Computer  microswitch  static bicycle’s pedaling  program | Improvement of skills (mobility) |
| Lancioni et al. 2017 [34] | Case study | Effectiveness | Italy  day care center | 11 | Unclear | Computer  microswitch  program | Improvement of skills |
|  |  |  |  | 10 | Unclear | Computer  microswitch  program | Improvement of skills |
| Lancioni et al. 2018a [35] | Case study | Effectiveness | Italy  day care center | 8 | Talking Alarm Clock app | App and  earpieces | Improvement of performing adls |
|  |  |  |  | 9 | Unclear | Walker  assistive technology | Improvement of skills (mobility) |
| Lancioni et al. 2018b [36] | Case study | Effectiveness and social validation | Italy  day care center | 10 | Unclear | Walker  assistive technology | Improvement of skills (mobility) |
| Lee et al. 2013 [37] | RCT | Effectiveness | China  home | 19 | CELP | Touch-screen notebook  program | Improvement of skills (memory) |
| Leuty et al. 2013[38] | Case study | Usability | Canada  Unclear | 6 | Engaging Platform for Art Development ePAD | Platform | Improvement of engagement |
| Liang et al. 2017 [39] | RCT | Effectiveness | New Zealand  day care center and home | 60 | PARO | Robot | Enabling reminiscence therapy and improvement of symptoms  and communication |
| Lindqvist et al. 2015 [40] | Qualitative study | Usability | Sweden  home | 30 (10 PwD) | Various | Assistive technology  various | Various |
| McEwen et al. 2014 [41] | Case study | Effectiveness and  feasibility | Canada  outpatient setting | 1 | Interactive rehabilitation exercise (IREX) software | Television  virtual reality | Improvement of skills (mobility) |
| Mervin et al. 2018 [42] | RCT | Cost-effectiveness | Australia  nursing Home | 415 | PARO | Robot | Enabling reminiscence therapy and improvement of symptoms  and communication |
| Mooney et al. 2018 [43] | Case study | Effectiveness | USA  home | 6 | CoChat | Tablet  app | Improvement of communication |
| Moyle et al. 2013 [44] | RCT | Effectiveness | Australia  nursing home | 18 | PARO | Robot | Enabling reminiscence therapy and improvement of symptoms  and communication |
| Moyle et al. 2016 [45] | Case study | Effectiveness and feasibility | Australia  nursing home | 5 | CuDDler | Robot | Enabling reminiscence therapy and improvement of symptoms  and communication |
| Moyle et al. 2017 [46] | RCT | Effectiveness | Australia  nursing home | 415 | PARO | Robot | Enabling reminiscence therapy and improvement of symptoms  and communication |
| Moyle et al. 2018 [47] | RCT | Effectiveness | Australia  nursing home | Day time analysis: 175 night time analysis: 280 | PARO | Robot | Enabling reminiscence therapy and improvement of symptoms  and communication |
| Nishiura et al. 2014 [48] | Case study | Feasibility | Japan  nursing home | 1 | PaPeRo | Robots | Improvement of engagement |
| Padala et al. 2017 [49] | RCT | Effectiveness | USA  home | 30 | Wii Fit | Console | Improvement of skills (mobility) |
| Perilli et al. 2013 [50] | Case study | Effectiveness and  social validation | Unclear  day care center | 5 | Unclear | Computer-aided telephone system | Enabling to make phone calls (communication) |
| Perugia et al. 2017 [51] | Study 1: exclusion intervention | Effectiveness | Spain  nursing home | 14 | Pleo | Robot | Enabling recreation and improvement of communication |
|  | Study 2: CT |  |  |  |  |  |  |
| Pringle und Somerville 2013 [52] | Case study | Effectiveness | UK  nursing Home | 8 | Unclear | Tablet  program | Enabling reminiscence therapy |
| Silva et al. 2017 [53] | RCT | Effectiveness | Portugal  home and day care center | 51 | SenseCam and MEMO+ | Monitoring camera | Improvement of skills (memory) |
| Takayanagi et al. 2014 [54] | CT | Effectiveness | Japan  nursing home | 30 | PARO | Robot | Enabling reminiscence therapy and improvement of symptoms  and communication |
| Tchalla et al. 2013 [55] | RCT | Effectiveness | France  home | 96 | HBTec-TS + various | Monitoring sensors and telephone | Improvement of care (preventing falls, fast reaction) |
| Tyack et al. 2017 [56] | Repeated measures study with mixed methods | Effectiveness | UK  home | 24 | Unclear | Tablet  app | Enabling reminiscence therapy |
| Valenti Soler et al. 2015 [57] | RCT | Effectiveness | Spain  nursing home | 101 (phase 1) and  110 (phase 2) | NAO + PARO | Robot | Various |
|  | Pre-post study |  | Spain  day care center | 20 (phase 1) and  17 (phase 2) |  |  |  |
| Wang et al. 2017 [58] | Qualitative study | Exploration | Canada  home (lab) | 20 | ED | Robot | Improvement of performing adls |
| Whelan et al. 2017 [59] | Case study | Effectiveness and  usability | Ireland  nursing home | 2 | MARIO | Robot | Improvement of communication and enabling reminiscence therapy and recreation |
| Woodberry et al. 2015 [60] | Case study | Effectiveness | UK  home | 6 | SenseCam | Monitoring camera | Improvement of skills (memory) |
| Yamazaki et al. 2014 [61] | Case study | Acceptability | Denmark  home | 1 | Telenoid R2 | Robot | Initiation of communication |
| Yasuda et al. 2013 [62] | Case study | Effectiveness | Japan  home | 4 | Unclear | Computer videophone | Enabling reminiscence therapy and improvement of communication |
|  |  |  |  |  | Unclear | Computer videophone  video | Improvement of engagement |
| Zmily et al. 2014 [63] | Case study | Usability | Jordan  nursing home | 10 | ADcope | App | Various |

# References

1. Abbate S, Avvenuti M, Light J. Usability Study of a Wireless Monitoring System among Alzheimer’s Disease Elderly Population. Int J Telemed Appl 2014;2014:617495. PMID:24963289

2. Astell AJ, Joddrell P, Groenewoud H, Lange J de, Goumans M, Cordia A, Schikhof Y. Does familiarity affect the enjoyment of touchscreen games for people with dementia? Int J Med Inform 2016;91:e1-8. PMID:26897552

3. Astell AJ, Smith SK, Potter S, Preston-Jones E. Computer Interactive Reminiscence and Conversation Aid groups-Delivering cognitive stimulation with technology. Alzheimers Dement (N Y) 2018;4(4):481-487. PMID:30258977

4. Bayen E, Jacquemot J, Netscher G, Agrawal P, Tabb Noyce L, Bayen A. Reduction in Fall Rate in Dementia Managed Care Through Video Incident Review: Pilot Study. J Med Internet Res 2017;19(10):e339. PMID:29042342

5. Begum M, Wang R, Huq R, Mihailidis A. Performance of daily activities by older adults with dementia: the role of an assistive robot. IEEE Int Conf Rehabil Robot 2013;2013:6650405. PMID:24187224

6. Begum M, Huq R, Wang R, Mihailidis A. Collaboration of an assistive robot and older adults with dementia. Gerontechnology 2015;13(4):405-419. doi:10.4017/gt.2015.13.4.005.00

7. Bejan A, Murko P, Müller N, König P, Kunze C. Using Surface Table Computers to Promote the Well-Being of People with Dementia. Stud Health Technol Inform 2017;242:23-26. PMID:28873771

8. Bejan A, Gündogdu R, Butz K, Müller N, Kunze C, König P. Using multimedia information and communication technology (ICT) to provide added value to reminiscence therapy for people with dementia : Lessons learned from three field studies. Z Gerontol Geriatr 2018;51(1):9-15. PMID:29218401

9. Bier N, Paquette G, Macoir J. Smartphone for smart living: Using new technologies to cope with everyday limitations in semantic dementia. Neuropsychol Rehabil 2018;28(5):734-754. PMID:26483262

10. Boyd H, Evans N, Cheston R, Noonan K, Harris N. Home Testing of a Digital Prompter for People with Dementia. Stud Health Technol Inform 2017;242:27-30. PMID:28873772

11. Burdea G, Polistico K, Krishnamoorthy A, House G, Rethage D, Hundal J, Damiani F, Pollack S. Feasibility study of the BrightBrainer™ integrative cognitive rehabilitation system for elderly with dementia. Disabil Rehabil Assist Technol 2015;10(5):421-432. PMID:24679074

12. Burdea GC, Polistico K, House GP, Liu RR, Muñiz R, Macaro NA, Slater LM. Novel integrative virtual rehabilitation reduces symptomatology of primary progressive aphasia—a case report. Int J Neurosci 2015;125(12):949-958. PMID:25485610

13. Cheong C-K, Lim K-H, Jang J-W, Jhoo JH. The effect of telemedicine on the duration of treatment in dementia patients. J Telemed Telecare 2015;21(4):214-218. PMID:25586813

14. Chu M-T, Khosla R, Khaksar SMS, Nguyen K. Service innovation through social robot engagement to improve dementia care quality. Assist Technol 2017;29(1):8-18. PMID:27064692

15. Cutler C, Hicks B, Innes A. Does digital gaming enable healthy aging for community-dwelling people with dementia? Games and Culture 2016;11(1):104-129. doi:10.1177/1555412015600580

16. Dethlefs N, Milders M, Cuayáhuitl H, Al-Salkini T, Douglas L. A natural language-based presentation of cognitive stimulation to people with dementia in assistive technology: A pilot study. Inform Health Soc Care 2017;42(4):349-360. PMID:28068146

17. Ehret S, Putze F, Miller-Teynor H, Kruse A, Schultz T. Technique-based game for daycare visitors with and without dementia: Effects, heuristics and correlates. Z Gerontol Geriatr 2017;50(1):35-44. PMID:27370267

18. Evans N, Cheston R, Harris N. Personal message cards: An evaluation of an alternative method of delivering simulated presence therapy. Dementia (London) 2016;15(6):1703-1715. PMID:25767142

19. Foloppe DA, Richard P, Yamaguchi T, Etcharry-Bouyx F, Allain P. The potential of virtual reality-based training to enhance the functional autonomy of Alzheimer’s disease patients in cooking activities: A single case study. Neuropsychol Rehabil 2018;28(5):709-733. PMID:26480838

20. Gustafsson C, Svanberg C, Müllersdorf M. Using a Robotic Cat in Dementia Care: A Pilot Study. J Gerontol Nurs 2015;41(10):46-56. PMID:26488255

21. Imbeault H, Langlois F, Bocti C, Gagnon L, Bier N. Can people with Alzheimer’s disease improve their day-to-day functioning with a tablet computer? Neuropsychol Rehabil 2018;28(5):779-796. PMID:28278593

22. Jentoft R, Holthe T, Arntzen C. The use of assistive technology in the everyday lives of young people living with dementia and their caregivers. Can a simple remote control make a difference? Int Psychogeriatr 2014;26(12):2011-2021. PMID:24932651

23. Jones C, Moyle W, Murfield J, Draper B, Shum D, Beattie E, Thalib L. Does Cognitive Impairment and Agitation in Dementia Influence Intervention Effectiveness? Findings From a Cluster-Randomized-Controlled Trial With the Therapeutic Robot, PARO. J Am Med Dir Assoc 2018;19(7):623-626. PMID:29656838

24. Kerssens C, Sattler M, Monteiro A. Managing dementia symptoms and needs using technology. J Gerontol Nurs 2014;40(7):16-20. PMID:24892310

25. Kerssens C, Kumar R, Adams AE, Knott CC, Matalenas L, Sanford JA, Rogers WA. Personalized technology to support older adults with and without cognitive impairment living at home. Am J Alzheimers Dis Other Deme 2015;30(1):85-97. PMID:25614507

26. Khosla R, Nguyen K, Chu M-T. Human Robot Engagement and Acceptability in Residential Aged Care. Int J Hum Comput Interact 2017;33(6):510-522. doi:10.1080/10447318.2016.1275435

27. Kouroupetroglou C, Casey D, Raciti M, Barrett E, D’Onofrio G, Ricciardi F, Giuliani F, Greco A, Sancarlo D, Mannion A, Whelan S, Pegman G, Koumpis A, Reforgiato Recupero D, Kouroupetroglou A, Santorelli A. Interacting with Dementia: The MARIO Approach. Stud Health Technol Inform 2017;242:38-47. PMID:28873774

28. Kuwamura K, Nishio S, Sato S. Can We Talk through a Robot As if Face-to-Face? Long-Term Fieldwork Using Teleoperated Robot for Seniors with Alzheimer’s Disease. Front Psychol 2016;7:1066. PMID:27486416

29. Lancioni GE, Perilli V, O’Reilly MF, Singh NN, Sigafoos J, Bosco A, Caffò AO, Picucci L, Cassano G, Groeneweg J. Technology-based orientation programs to support indoor travel by persons with moderate Alzheimer’s disease: impact assessment and social validation. Res Dev Disabil 2013;34(1):286-293. PMID:22982468

30. Lancioni GE, Singh NN, O’Reilly MF, Sigafoos J, Renna C, Ventrella M, Pinto K, Minervini MG, Oliva D, Groeneweg J. Supporting daily activities and indoor travel of persons with moderate Alzheimer’s disease through standard technology resources. Res Dev Disabil 2013;34(8):2351-2359. PMID:23711628

31. Lancioni GE, Caro MF, Singh NN, O’Reilly MF, Sigafoos J, Vanna F, Spinelli C, Santoruvo A, Buono S, Trubia G. Further evaluation of programs for promoting daily activities and indoor orientation and travel in persons with moderate Alzheimer’s disease. Life Span Disabil 2014;17(2):245-263.

32. Lancioni GE, Singh NN, O’Reilly MF, Sigafoos J, Renna C, Pinto K, Vanna F de, Caffò AO, Stasolla F. Persons with moderate Alzheimer’s disease use simple technology aids to manage daily activities and leisure occupation. Res Dev Disabil 2014;35(9):2117-2128. PMID:24881006

33. Lancioni GE, Singh NN, O’Reilly MF, Sigafoos J, D’Amico F, Sasanelli G, Vanna F de, Signorino M. Persons with Alzheimer’s disease engage in leisure and mild physical activity with the support of technology-aided programs. Res Dev Disabil 2015;37:55-63. PMID:25460220

34. Lancioni GE, Singh NN, O’Reilly MF, Sigafoos J, D’Amico F, Pinto K, Chiapparino C. Supporting Simple Activity Engagement in Persons With Moderate to Severe Alzheimer’s Disease Through a Technology-Aided Program. Am J Alzheimers Dis Other Deme 2017;32(3):137-144. PMID:28423935

35. Lancioni GE, Singh NN, O’Reilly MF, Sigafoos J, D’Amico F, Laporta D, Cattaneo MG, Scordamaglia A, Pinto K. Technology-Based Behavioral Interventions for Daily Activities and Supported Ambulation in People With Alzheimer’s Disease. Am J Alzheimers Dis Other Deme 2018;33(5):318-326. PMID:29742903

36. Lancioni GE, Singh NN, O’Reilly MF, Sigafoos J, D’Amico F, Pinto K, Vanna F de, Caffò AO. Promoting supported ambulation in persons with advanced Alzheimer’s disease: a pilot study. Disabil Rehabil Assist Technol 2018;13(1):101-106. PMID:28287045

37. Lee GY, Yip CCK, Yu ECS, Man DWK. Evaluation of a computer-assisted errorless learning-based memory training program for patients with early Alzheimer’s disease in Hong Kong: a pilot study. Clin Interv Aging 2013;8:623-633. PMID:23766638

38. Leuty V, Boger J, Young L, Hoey J, Mihailidis A. Engaging older adults with dementia in creative occupations using artificially intelligent assistive technology. Assist Technol 2013;25(2):72-79. PMID:23923689

39. Liang A, Piroth I, Robinson H, MacDonald B, Fisher M, Nater UM, Skoluda N, Broadbent E. A Pilot Randomized Trial of a Companion Robot for People With Dementia Living in the Community. J Am Med Dir Assoc 2017;18(10):871-878. PMID:28668664

40. Lindqvist E, Larsson TJ, Borell L. Experienced usability of assistive technology for cognitive support with respect to user goals. NeuroRehabilitation 2015;36(1):135-149. PMID:25547777

41. McEwen D, Taillon-Hobson A, Bilodeau M, Sveistrup H, Finestone H. Two-week virtual reality training for dementia: Single case feasibility study. J Rehabil Res Dev 2014;51(7):1069-1076. PMID:25437527

42. Mervin MC, Moyle W, Jones C, Murfield J, Draper B, Beattie E, Shum DHK, O’Dwyer S, Thalib L. The Cost-Effectiveness of Using PARO, a Therapeutic Robotic Seal, to Reduce Agitation and Medication Use in Dementia: Findings from a Cluster-Randomized Controlled Trial. J Am Med Dir Assoc 2018;19(7):619-622.e1. PMID:29325922

43. Mooney A, Bedrick S, Noethe G, Spaulding S, Fried-Oken M. Mobile technology to support lexical retrieval during activity retell in primary progressive aphasia. Aphasiology 2018;32(6):666-692. doi:10.1080/02687038.2018.1447640

44. Moyle W, Cooke M, Beattie E, Jones C, Klein B, Cook G, Gray C. Exploring the effect of companion robots on emotional expression in older adults with dementia: a pilot randomized controlled trial. J Gerontol Nurs 2013;39(5):46-53. PMID:23506125

45. Moyle W, Jones C, Sung B, Bramble M, O’Dwyer S, Blumenstein M, Estivill-Castro V. What Effect Does an Animal Robot Called CuDDler Have on the Engagement and Emotional Response of Older People with Dementia? A Pilot Feasibility Study. Int J of Soc Robotics 2016;8:145-156. doi:10.1007/s12369-015-0326-7

46. Moyle W, Jones CJ, Murfield JE, Thalib L, Beattie ERA, Shum DKH, O’Dwyer ST, Mervin MC, Draper BM. Use of a Robotic Seal as a Therapeutic Tool to Improve Dementia Symptoms: A Cluster-Randomized Controlled Trial. J Am Med Dir Assoc 2017;18(9):766-773. PMID:28780395

47. Moyle W, Jones C, Murfield J, Thalib L, Beattie E, Shum D, O’Dwyer S, Mervin MC, Draper B. Effect of a robotic seal on the motor activity and sleep patterns of older people with dementia, as measured by wearable technology: A cluster-randomised controlled trial. Maturitas 2018;110:10-17. PMID:29563027

48. Nishiura Y, Inoue T, Nihei M. Appropriate talking pattern of an information support robot for people living with dementia: a case study. J Assist Technol 2014;8(4):177-187. doi:10.1108/JAT-12-2013-0035

49. Padala KP, Padala PR, Lensing SY, Dennis RA, Bopp MM, Roberson PK, Sullivan DH. Home-Based Exercise Program Improves Balance and Fear of Falling in Community-Dwelling Older Adults with Mild Alzheimer’s Disease: A Pilot Study. J Alzheimers Dis 2017;59(2):565-574. PMID:28655135

50. Perilli V, Lancioni GE, Laporta D, Paparella A, Caffò AO, Singh NN, O’Reilly MF, Sigafoos J, Oliva D. A computer-aided telephone system to enable five persons with Alzheimer’s disease to make phone calls independently. Res Dev Disabil 2013;34(6):1991-1997. PMID:23584179

51. Perugia G, Diaz Doladeras M, Mallofre AC, Rauterberg M, Barakova E. Modelling engagement in dementia through behaviour. Contribution for socially interactive robotics. IEEE Int Conf Rehabil Robot 2017;2017:1112-1117. PMID:28813970

52. Pringle A, Somerville S. Computer-assisted reminiscence therapy: developing practice. Mental Health Practice 2013;17(4):34-37. doi:10.7748/mhp2013.12.17.4.34.e830

53. Silva AR, Pinho MS, Macedo L, Moulin CJA. The Cognitive Effects of Wearable Cameras in Mild Alzheimer Disease - An Experimental Study. Curr Alzheimer Res 2017;14(12):1270-1282. PMID:28558637

54. Takayanagi K, Kirita T, Shibata T. Comparison of Verbal and Emotional Responses of Elderly People with Mild/Moderate Dementia and Those with Severe Dementia in Responses to Seal Robot, PARO. Front Aging Neurosci 2014;6:257. PMID:25309434

55. Tchalla AE, Lachal F, Cardinaud N, Saulnier I, Rialle V, Preux P-M, Dantoine T. Preventing and managing indoor falls with home-based technologies in mild and moderate Alzheimer’s disease patients: pilot study in a community dwelling. Dement Geriatr Cogn Disord 2013;36(3-4):251-261. PMID:23949277

56. Tyack C, Camic PM, Heron MJ, Hulbert S. Viewing Art on a Tablet Computer: A Well-Being Intervention for People With Dementia and Their Caregivers. J Appl Gerontol 2017;36(7):864-894. PMID:26675353

57. Valentí Soler M, Agüera-Ortiz L, Olazarán Rodríguez J, Mendoza Rebolledo C, Pérez Muñoz A, Rodríguez Pérez I, Osa Ruiz E, Barrios Sánchez A, Herrero Cano V, Carrasco Chillón L, Felipe Ruiz S, López Alvarez J, León Salas B, Cañas Plaza JM, Martín Rico F, Abella Dago G, Martínez Martín P. Social robots in advanced dementia. Front Aging Neurosci 2015;7:133. PMID:26388764

58. Wang RH, Sudhama A, Begum M, Huq R, Mihailidis A. Robots to assist daily activities: views of older adults with Alzheimer’s disease and their caregivers. Int Psychogeriatr 2017;29(1):67-79. PMID:27660047

59. Whelan S, Kouroupetroglou C, Santorelli A, Raciti M, Barrett E, Casey D. Investigating the Effect of Social Robot Embodiment. Stud Health Technol Inform 2017;242:523-526. PMID:28873848

60. Woodberry E, Browne G, Hodges S, Watson P, Kapur N, Woodberry K. The use of a wearable camera improves autobiographical memory in patients with Alzheimer’s disease. Memory 2015;23(3):340-349. PMID:24528204

61. Yamazaki R, Nishio S, Ishiguro H, Nørskov M, Ishiguro N, Balistreri G. Acceptability of a Teleoperated Android by Senior Citizens in Danish Society. Int J of Soc Robotics 2014;6(3):429-442. doi:10.1007/s12369-014-0247-x

62. Yasuda K, Kuwahara N, Kuwabara K, Morimoto K, Tetsutani N. Daily assistance for individuals with dementia via videophone. Am J Alzheimers Dis Other Deme 2013;28(5):508-516. PMID:23813611

63. Zmily A, Mowafi Y, Mashal E. Study of the usability of spaced retrieval exercise using mobile devices for Alzheimer’s disease rehabilitation. JMIR Mhealth Uhealth 2014;2(3):e31. PMID:25124077
