# Supplementary material for: Technology-Assisted Home Care for People With Dementia and Their Relatives: Scoping Review
Source: JMIR Aging. 2021 Jan 20;4(1):e25307. doi: 10.2196/25307 (PMC7857954; doi:10.2196/25307)
Supplement: Multimedia Appendix 3 [file aging_v4i1e25307_app3.docx]

**Appendix 3: Study details – informal carers**

| **Author** | **Study design** | **Study aim** | **Country**  **Setting** | **Number of participants** | **Technology name** | **Technology group** | **Technology aim** |
| --- | --- | --- | --- | --- | --- | --- | --- |
| Au et al. 2014 [1] | RCT | Effectiveness | China  home | 60 | TAPES | Telephone | Improvement of knowledge and symptoms |
| Au et al. 2015 [2] | RCT | Effectiveness | China  home | 62 | Unclear | Telephone | Improvement of knowledge and symptoms |
| Au 2015 [3] | RCT | Effectiveness | China  home | 96 | Unclear | Telephone | Improvement of symptoms |
| Austrom et al. 2015 [4] | Pre-post study with  mixed methods | Effectiveness | USA  home | 5 | Unclear | Telehealth  videoconference | Improvement of knowledge and symptoms |
| Bateman et al. 2017 [5] | Pre-post study with  mixed methods | Feasibility | USA  home | 6 | Facebook group | Facebook group | Improvement of symptoms and care |
| Berwig et al. 2017 [6] | RCT | Effectiveness | Germany  home | 92 | Reach II | Telephone | Improvement of symptoms, knowledge and care |
| Blom et al. 2015 [7] | RCT | Effectiveness | Netherlands  home | 251 | Mastery over Dementia | Internet course | Improvement of symptoms |
| Boessen et al. 2017 [8] | Cross sectional study with  mixed methods | Use and usability | Netherland  home | 7 | Unclear | Platform | Improvement of caregiver`s network |
| Boots et al. 2018 [9] | RCT | Effectiveness | Netherlands  home | 81 | Partner in Balance | Internet course | Improvement of symptoms |
| Boyd et al. 2014 [10] | Case study | Usability | Ireland  day care center | 5 | STAR Training Website | Web portal | Improvement of knowledge and care |
| Brown et al. 2016 [11] | Quasiexperimental study with  mixed methods | Feasibility | USA  home | 22 | CareHeroes | Smartphone  app | Improvement of knowledge and care |
| Chodosh et al. 2015 [12] | RCT | Effectiveness and costs | USA  home | 302 | Unclear | Telephone and mail | Improvement of symptoms, knowledge and care |
| Cristancho-Lacroix et al. 2015 [13] | Pilot RCT | Effectiveness and acceptability | France  home | 49 | Diapason | Internet course | Improvement of symptoms, knowledge and care |
| Czaja et al. 2013 [14] | RCT | Feasibility and efficacy | USA  home | 110 | REACHII | Videophone | Improvement of symptoms, knowledge and care |
| Czaja et al. 2018 [15] | RCT | Effectiveness | USA  home | 146 | Community REACH | Telephone | Improvement of symptoms, knowledge and care |
| Dam et al. 2017 [16] | Pre-post study | Feasibility and effectiveness | Netherland  home | 25 | Inlife | Platform | Improvement of symptoms and care |
| Damianakis et al. 2018 [17] | Qualitative study | Exploration | Canada  home | 24 | Caring for Others | Virtual online support group | Improvement of symptoms and care |
| Davis et al. 2014 [18] | Pre-post study | Development | USA  home | 5 | Story-Call | Smartphone  app | Improvement of symptoms and care |
| Davis et al. 2015 [19] | Pre-post study | Proof-of-concept | USA  home | 5 | Story-Call | Smartphone  app | Improvement of symptoms and care |
| Dowling et al. 2014 [20] | Pilot RCT | Feasibility | USA  home | 24 | Life Enhancing Activities for Family Caregivers | Telehealth  internet course | Improvement of symptoms |
| Easom et al. 2013 [21] | Pre-post study | Effectiveness | USA  home | 85 | GA REACH | Telephone | Improvement of symptoms, knowledge and care |
| Fowler et al. 2016a [22] | Cross sectional study | Use and usability | USA  home | 28 | Virtual Neighborhood (VHN) | Website | Improvement of knowledge and care |
|  | Case study |  |  | 2 |  |  |  |
| Fowler et al. 2016b [23] | RCT | Effectiveness | USA  home | 28 | Virtual Neighborhood (VHN) | Website | Improvement of knowledge and care |
| Garzon-Maldonado et al. 2017 [24] | CT | Evaluation of costs | Spain  home | 194 | Telephone Assistance System (TAS) | Telephone | Improvement of symptoms and care |
| Gaugler et al. 2015 [25] | Pre-post study | Effectiveness | USA  home | 41 | CARES, Dementia Care for Families | Internet course | Improvement of symptoms, knowledge and care |
| Gaugler et al. 2016 [26] | Cross sectional study with mixed method | Feasibility and utility | USA  home | 30 | Care to Plan | Online tool | Improvement of care |
| Graham‐Phillips et al. 2016 [27] | RCT | Exploration | USA  home | 323 | REACH II | Telephone | Improvement of symptoms, knowledge and care |
| Griffiths et al. 2016 [28] | Pre-post study | Fidelity and efficacy | USA  home | 30 | Tele-Savvy Caregiver Program | Tele-conferences and internet course | Improvement of symptoms and care |
| Griffiths et al. 2018 [29] | Pre-post study | Feasibility and efficacy | USA  home | 64 | Tele-Savvy Caregiver Program | Tele-conferences and internet course | Improvement of symptoms and care |
| Hattink et al. 2015 [30] | RCT | Effectiveness,  userfriendliness and usefulness | Netherlands, UK  home | 142 | Web-Based STAR E-Learning Course | Internet course | Improvement of symptoms, knowledge and care |
| Hicken et al. 2017 [31] | RCT | Effectiveness | USA  home | 229 | Unclear | Internet course | Improvement of symptoms, knowledge and care |
| Horne et al. 2018 [32] | Qualitative study | Usefulness | Australia  home | 16 | Managing Medicines for People with Dementia | Website | Improvement of knowledge |
| Kajiyama et al. 2013 [33] | RCT | Effectiveness | USA  home | 150 | iCare program | Internet course | Improvement of symptoms |
| Kajiyama et al. 2018 [34] | Pre-post study | Development and evaluation | USA  home | 25 | Webnovela Mirella | Webnovela | Improvement of symptoms |
| Killin et al. 2018 [35] | Qualitative  study | Feasibility | UK  home | 20 | Digital Support Platform (DSP) | Platform | Improvement of knowledge and care |
| Knippenberg et al. 2017 [36] | Case series | Feasibility | Netherlands  home | 31 | PsyMate  as medium | Touchscreen device | Provide assessment of symptoms |
| Kuo et al. 2017 [37] | RCT | Effectiveness | Taiwan  home | 116 | Unclear | Telephone | Improvement of symptoms |
| Kwok et al. 2013 [38] | RCT | Effectiveness | China  home | 38 | Unclear | Telephone | Improvement of symptoms, knowledge and care |
| Kwok et al. 2014 [39] | Pre-post study | Effectiveness | China  home | 36 | Unclear | Website | Improvement of symptoms and care |
| Lai et al. 2013 [40] | Pilot CT | Effectiveness | China  home | 11 | Website “Ginkgo”  as medium | Internet course | Improvement of symptoms, knowledge and care |
| Lundberg 2014 [41] | Case study with  mixed methods | Exploration | Sweden  home | 10 households | ACTION | ICT | Improvement of symptoms, knowledge and care |
| Martindale-Adams et al. 2013 [42] | RCT | Effectiveness | USA  home | 154 | CONNECT | Telephone | Improvement of symptoms, knowledge and care |
| Matthews et al. 2015 [43] | Pre-post study with  mixed methods | Usability | USA  home | 18 | Unclear | Monitoring  camera | Improvement of symptoms and care |
| Mavandadi et al. 2017a [44] | CT | Effectiveness | USA  home | 440 | Unclear | Telephone | Improvement of symptoms and care |
| Mavandadi et al. 2017b [45] | Pilot RCT | Effectiveness | USA  home | 75 | Unclear | Telephone | Improvement of symptoms and care |
| McKechnie et al. 2014 [46] | Pre-post study with mixed methods | Effectiveness | UK  home | 128 | Talking Point | Internet support forum | Improvement of symptoms, knowledge and care |
| Nauha et al. 2018 [47] | Case study | Usefulness and usability | Finland  home | 3 | Various | Various | Improvement of care |
| Nunez-Naveira et al. 2016 [48] | Pilot RCT | Effectiveness | Denmark, Poland, Spain  home | 61 | UnderstAID | Platform | Improvement of symptoms, knowledge and care |
| O’Connell et al. 2014 [49] | Case study | Effectiveness | Canada  home | 11 | Unclear | Telehealth  videoconference | Improvement of symptoms and care |
| O’Connor et al. 2014 [50] | Case study | Feasibility | USA  home | 10 | Second Life platform | Virtual reality | Improvement of symptoms |
| Pagán-Ortiz et al. 2014 [51] | RCT | Effectiveness | Puerto Rico, Mexico, USA  home | 40 | Cuidatecuidador.com | Website | Improvement of symptoms, knowledge and care |
| Pot et al. 2015 [52] | Secondary data analysis | Acceptability | Netherlands  home | 149 | Mastery over Dementia | Internet course | Improvement of symptoms |
| Schaller et al. 2015 [53] | Qualitative study | Exploration | Germany  home | 42 | eHealthMonitor Dementia Portal | Web portal | Improvement of symptoms, knowledge and care |
| Schaller et al. 2016 [54] | Pre-post study with mixed methods | Usefulness and effectiveness | Germany  home | 25 | eHealthMonitor Dementia Portal | Web portal | Improvement of symptoms, knowledge and care |
| Scharett et al. 2017 [55] | Cross sectional study | Exploration of the content | Unclear | 500  posts | ALZConnected.org | Online peer support group | Improvement of symptoms, knowledge and care |
| Schinköthe und Wilz 2014 [56] | Secondary data analysis | Assessment testing | Germany  home | 45 | TeleTAnDem | Telephone | Improvement of symptoms |
| Schinköthe et al. 2015 [57] | Secondary data analysis | Evaluation | Germany  home | 43 | TeleTAnDem | Telephone | Improvement of symptoms |
| Steffen und Gant 2016 [58] | RCT | Effectiveness | USA  home | 74 | Unclear | Telephone | Improvement of symptoms and care |
| Torkamani et al. 2014 [59] | Pilot RCT | Effectiveness | UK, Spain, Greece  home | 60 | ALADDIN | Platform | Improvement of symptoms, knowledge, care and communication with clinicals |
| Tremont et al. 2015 [60] | RCT | Effectiveness | USA  home | 250 | Telephone Tracking–Caregiver (FITT-C) | Telephone | Improvement of symptoms, knowledge and care |
| Tremont et al. 2017 [61] | RCT | Cost-effectiveness | USA  home | 250 | Telephone Tracking–Caregiver (FITT-C) | Telephone | Improvement of symptoms, knowledge and care |
| van Mierlo et al. 2015 [62] | RCT | Effectiveness and user-friendliness | Netherland  home | 73 | DEM-DISC | ICT | Improvement of knowledge and care |
| Williams et al. 2013 [63] | Case study | Feasibility | USA  home | 2 | Unclear | Telehealth  monitoring | Improvement of symptoms and care |
| Wilz and Soellner 2016 [64] | RCT | Effectiveness | Germany  home | 229 | TeleTAnDem | Telephone | Improvement of symptoms |
| Wilz et al. 2017 [65] | RCT  follow up data, cross sectional | Evaluation of long-term effects | Germany  home | 105 | TeleTAnDem | Telephone | Improvement of symptoms |
| Wilz et al. 2018a [66] | RCT | Effectiveness | Germany  home | 273 | TeleTAnDem | Telephone | Improvement of symptoms |
| Wilz et al. 2018b [67] | Secondary data analysis | Evaluation | Germany  home | 139 | TeleTAnDem | Telephone | Improvement of symptoms |

References

1. Au A, Wong MK, Leung LM, Leung P, Wong A. Telephone-assisted pleasant-event scheduling to enhance well-being of caregivers of people with dementia: a randomised controlled trial. Hong Kong Med J 2014;20(3 Suppl 3):30-33. PMID:25001033

2. Au A, Gallagher-Thompson D, Wong M-K, Leung J, Chan W-C, Chan CC, Lu H-J, Lai MK, Chan K. Behavioral activation for dementia caregivers: scheduling pleasant events and enhancing communications. Clin Interv Aging 2015;10:611-619. PMID:25848237

3. Au A. Developing Volunteer-Assisted Behavioral Activation Teleprograms to Meet the Needs of Chinese Dementia Caregivers. Clin Gerontol 2015;38(3):190-202. doi:10.1080/07317115.2015.1008118

4. Austrom MG, Geros KN, Hemmerlein K, McGuire SM, Gao S, Brown SA, Callahan CM, Clark DO. Use of a multiparty web based videoconference support group for family caregivers: Innovative practice. Dementia (London) 2015;14(5):682-690. PMID:25062788

5. Bateman DR, Brady E, Wilkerson D, Yi E-H, Karanam Y, Callahan CM. Comparing Crowdsourcing and Friendsourcing: A Social Media-Based Feasibility Study to Support Alzheimer Disease Caregivers. JMIR Res Protoc 2017;6(4):e56. PMID:28396304

6. Berwig M, Heinrich S, Spahlholz J, Hallensleben N, Brähler E, Gertz H-J. Individualized support for informal caregivers of people with dementia - effectiveness of the German adaptation of REACH II. BMC Geriatr 2017;17(1):286. PMID:29233097

7. Blom MM, Zarit SH, Groot Zwaaftink RBM, Cuijpers P, Pot AM. Effectiveness of an Internet intervention for family caregivers of people with dementia: results of a randomized controlled trial. PLoS ONE 2015;10(2):e0116622. PMID:25679228

8. Boessen ABCG, Verwey R, Duymelinck S, van Rossum E. An Online Platform to Support the Network of Caregivers of People with Dementia. J Aging Res 2017;2017:3076859. PMID:28894609

9. Boots LM, Vugt ME de, Kempen GI, Verhey FR. Effectiveness of a Blended Care Self-Management Program for Caregivers of People With Early-Stage Dementia (Partner in Balance): Randomized Controlled Trial. J Med Internet Res 2018;20(7):e10017. PMID:30006327

10. Boyd K, Nugent C, Donnelly M, Bond R, Sterritt R, Hartin P. An investigation into the usability of the STAR training and re-skilling website for carers of persons with dementia. Annu Int Conf IEEE Eng Med Biol Soc 2014;2014:4139-4142. PMID:25570903

11. Brown EL, Ruggiano N, Page TF, Roberts L, Hristidis V, Whiteman KL, Castro J. CareHeroes Web and Android™ Apps for Dementia Caregivers: A Feasibility Study. Res Gerontol Nurs 2016;9(4):193-203. PMID:29977440

12. Chodosh J, Colaiaco BA, Connor KI, Cope DW, Liu H, Ganz DA, Richman MJ, Cherry DL, Blank JM, Del Carbone RP, Wolf SM, Vickrey BG. Dementia Care Management in an Underserved Community: The Comparative Effectiveness of Two Different Approaches. J Aging Health 2015;27(5):864-893. PMID:25656074

13. Cristancho-Lacroix V, Wrobel J, Cantegreil-Kallen I, Dub T, Rouquette A, Rigaud A-S. A web-based psychoeducational program for informal caregivers of patients with Alzheimer’s disease: a pilot randomized controlled trial. J Med Internet Res 2015;17(5):e117. PMID:25967983

14. Czaja SJ, Loewenstein D, Schulz R, Nair SN, Perdomo D. A videophone psychosocial intervention for dementia caregivers. Am J Geriatr Psychiatry 2013;21(11):1071-1081. PMID:23831174

15. Czaja SJ, Lee CC, Perdomo D, Loewenstein D, Bravo M, Moxley JH, Schulz R. Community REACH: An Implementation of an Evidence-Based Caregiver Program. Gerontologist 2018;58(2):e130-e137. PMID:29562361

16. Dam AEH, van Boxtel, Martin P. J., Rozendaal N, Verhey FRJ, Vugt ME de. Development and feasibility of Inlife: A pilot study of an online social support intervention for informal caregivers of people with dementia. PLoS ONE 2017;12(9):e0183386. PMID:28886056

17. Damianakis T, Wilson K, Marziali E. Family caregiver support groups: spiritual reflections’ impact on stress management. Aging Ment Health 2018;22(1):70-76. PMID:27647207

18. Davis BH, Nies MA, Shehab M, Shenk D. Developing a pilot e-mobile app for dementia caregiver support: Lessons learned. Online J Nurs Inform 2014;18(1):21-28.

19. Davis BH, Shehab M, Shenk D, Nies M. E-mobile pilot for community-based dementia caregivers identifies desire for security. Gerontechnology 2014;13(3):332-336. doi:10.4017/gt.2015.13.3.003.00

20. Dowling GA, Merrilees J, Mastick J, Chang VY, Hubbard E, Moskowitz JT. Life enhancing activities for family caregivers of people with frontotemporal dementia. Alzheimer Dis Assoc Disord 2014;28(2):175-181. PMID:24113564

21. Easom L, Alston G, Coleman R. A Rural Community Translation of a Dementia Caregiving Intervention. Online J Rural Nurs Health Care 2013;13(1):66-91. doi:10.14574/ojrnhc.v13i1.248

22. Fowler CN, Haney T, Lemaster M. Helping Dementia Caregivers Through Technology. Home Healthc Now 2016;34(4):203-209. PMID:27023296

23. Fowler CN, Kott K, Wicks MN, Rutledge C. An Interprofessional Virtual Healthcare Neighborhood: Effect on Self-Efficacy and Sleep Among Caregivers of Older Adults With Dementia. J Gerontol Nurs 2016;42(11):39-47. PMID:27598270

24. Garzón-Maldonado FJ, Gutiérrez-Bedmar M, Serrano-Castro V, Requena-Toro MV, Padilla-Romero L, García-Casares N. An assessesment of telephone assistance systems for caregivers of patients with Alzheimer’s disease. Neurologia 2017;32(9):595-601. PMID:27293022

25. Gaugler JE, Hobday JV, Robbins JC, Barclay MP. CARES® Dementia Care for Families™: Effects of Online, Psychoeducational Training on Knowledge of Person-Centered Care and Satisfaction. J Gerontol Nurs 2015;41(10):18-24. PMID:26270065

26. Gaugler JE, Reese M, Tanler R. Care to Plan: An Online Tool That Offers Tailored Support to Dementia Caregivers. Gerontologist 2016;56(6):1161-1174. PMID:26603183

27. Graham-Phillips A, Roth DL, Huang J, Dilworth-Anderson P, Gitlin LN. Racial and Ethnic Differences in the Delivery of the Resources for Enhancing Alzheimer’s Caregiver Health II Intervention. J Am Geriatr Soc 2016;64(8):1662-1667. PMID:27294873

28. Griffiths PC, Whitney MK, Kovaleva M, Hepburn K. Development and Implementation of Tele-Savvy for Dementia Caregivers: A Department of Veterans Affairs Clinical Demonstration Project. Gerontologist 2016;56(1):145-154. PMID:26566806

29. Griffiths PC, Kovaleva M, Higgins M, Langston AH, Hepburn K. Tele-Savvy: An Online Program for Dementia Caregivers. Am J Alzheimers Dis Other Deme 2018;33(5):269-276. PMID:29544342

30. Hattink B, Meiland F, van der Roest H, Kevern P, Abiuso F, Bengtsson J, Giuliano A, Duca A, Sanders J, Basnett F, Nugent C, Kingston P, Dröes R-M. Web-Based STAR E-Learning Course Increases Empathy and Understanding in Dementia Caregivers: Results from a Randomized Controlled Trial in the Netherlands and the United Kingdom. J Med Internet Res 2015;17(10):e241. PMID:26519106

31. Hicken BL, Daniel C, Luptak M, Grant M, Kilian S, Rupper RW. Supporting Caregivers of Rural Veterans Electronically (SCORE). J Rural Health 2017;33(3):305-313. PMID:27437642

32. Horne F, Burns P, Traynor V, Gillespie R, Mullan J, Baker A, Harrison L, Win KT. Managing medications for individuals living with a dementia: Evaluating a web-based information resource for informal carers. Int J Older People Nurs 2018;13(3):e12198. PMID:29745042

33. Kajiyama B, Thompson LW, Eto-Iwase T, Yamashita M, Di Mario J, Marian Tzuang Y, Gallagher-Thompson D. Exploring the effectiveness of an internet-based program for reducing caregiver distress using the iCare Stress Management e-Training Program. Aging Ment Health 2013;17(5):544-554. PMID:23461355

34. Kajiyama B, Fernandez G, Carter EA, Humber MB, Thompson LW. Helping Hispanic Dementia Caregivers Cope with Stress Using Technology-based Resources. Clin Gerontol 2018;41(3):209-216. PMID:29236621

35. Killin LOJ, Russ TC, Surdhar SK, Yoon Y, McKinstry B, Gibson G, MacIntyre DJ. Digital Support Platform: a qualitative research study investigating the feasibility of an internet-based, postdiagnostic support platform for families living with dementia. BMJ Open 2018;8(4):e020281. PMID:29654028

36. van Knippenberg RJM, Vugt ME de, Ponds RW, Myin-Germeys I, van Twillert B, Verhey FRJ. Dealing with daily challenges in dementia (deal-id study): an experience sampling study to assess caregiver functioning in the flow of daily life. Int J Geriatr Psychiatry 2017;32(9):949-958. PMID:27464472

37. Kuo L-M, Huang H-L, Liang J, Kwok Y-T, Hsu W-C, Su P-L, Shyu Y-IL. A randomized controlled trial of a home-based training programme to decrease depression in family caregivers of persons with dementia. J Adv Nurs 2017;73(3):585-598. PMID:27653753

38. Kwok T, Wong B, Ip I, Chui K, Young D, Ho F. Telephone-delivered psychoeducational intervention for Hong Kong Chinese dementia caregivers: a single-blinded randomized controlled trial. Clin Interv Aging 2013;8:1191-1197. PMID:24072965

39. Kwok T, Au A, Wong B, Ip I, Mak V, Ho F. Effectiveness of online cognitive behavioral therapy on family caregivers of people with dementia. Clin Interv Aging 2014;9:631-636. PMID:24748781

40. Lai CKY, Wong LF, Liu K-H, Lui W, Chan MF, Yap LSY. Online and onsite training for family caregivers of people with dementia: results from a pilot study. Int J Geriatr Psychiatry 2013;28(1):107-108. PMID:23225697

41. Lundberg S. The results from a two-year case study of an information and communication technology support system for family caregivers. Disabil Rehabil Assist Technol 2014;9(4):353-358. PMID:23855387

42. Martindale-Adams J, Nichols LO, Burns R, Graney MJ, Zuber J. A trial of dementia caregiver telephone support. Can J Nurs Res 2013;45(4):30-48. PMID:24617278

43. Matthews JT, Lingler JH, Campbell GB, Hunsaker AE, Hu L, Pires BR, Hebert M, Schulz R. Usability of a Wearable Camera System for Dementia Family Caregivers. J Healthc Eng 2015;6(2):213-238. PMID:26288888

44. Mavandadi S, Wray LO, DiFilippo S, Streim J, Oslin D. Evaluation of a Telephone-Delivered, Community-Based Collaborative Care Management Program for Caregivers of Older Adults with Dementia. Am J Geriatr Psychiatry 2017;25(9):1019-1028. PMID:28433550

45. Mavandadi S, Wright EM, Graydon MM, Oslin DW, Wray LO. A randomized pilot trial of a telephone-based collaborative care management program for caregivers of individuals with dementia. Psychol Serv 2017;14(1):102-111. PMID:28134558

46. McKechnie V, Barker C, Stott J. The effectiveness of an Internet support forum for carers of people with dementia: a pre-post cohort study. J Med Internet Res 2014;16(2):e68. PMID:24583789

47. Nauha L, Keränen NS, Kangas M, Jämsä T, Reponen J. Assistive technologies at home for people with a memory disorder. Dementia (London) 2018;17(7):909-923. PMID:27765896

48. Núñez-Naveira L, Alonso-Búa B, Labra C de, Gregersen R, Maibom K, Mojs E, Krawczyk-Wasielewska A, Millán-Calenti JC. UnderstAID, an ICT Platform to Help Informal Caregivers of People with Dementia: A Pilot Randomized Controlled Study. Biomed Res Int 2016;2016:5726465. PMID:28116300

49. O’Connell ME, Crossley M, Cammer A, Morgan D, Allingham W, Cheavins B, Dalziel D, Lemire M, Mitchell S, Morgan E. Development and evaluation of a telehealth videoconferenced support group for rural spouses of individuals diagnosed with atypical early-onset dementias. Dementia (London) 2014;13(3):382-395. PMID:24339063

50. O’Connor M-F, Arizmendi BJ, Kaszniak AW. Virtually supportive: a feasibility pilot study of an online support group for dementia caregivers in a 3D virtual environment. J Aging Stud 2014;30:87-93. PMID:24984911

51. Pagán-Ortiz ME, Cortés DE, Rudloff N, Weitzman P, Levkoff S. Use of an online community to provide support to caregivers of people with dementia. J Gerontol Soc Work 2014;57(6-7):694-709. PMID:24689359

52. Pot AM, Blom MM, Willemse BM. Acceptability of a guided self-help Internet intervention for family caregivers: mastery over dementia. Int Psychogeriatr 2015;27(8):1343-1354. PMID:25648589

53. Schaller S, Marinova-Schmidt V, Gobin J, Criegee-Rieck M, Griebel L, Engel S, Stein V, Graessel E, Kolominsky-Rabas PL. Tailored e-Health services for the dementia care setting: a pilot study of ‘eHealthMonitor’. BMC Med Inform Decis Mak 2015;15:58. PMID:26215731

54. Schaller S, Marinova-Schmidt V, Setzer M, Kondylakis H, Griebel L, Sedlmayr M, Graessel E, Maler JM, Kirn S, Kolominsky-Rabas PL. Usefulness of a Tailored eHealth Service for Informal Caregivers and Professionals in the Dementia Treatment and Care Setting: The eHealthMonitor Dementia Portal. JMIR Res Protoc 2016;5(2):e47. PMID:27050401

55. Scharett E, Madathil KC, Lopes S, Rogers H, Agnisarman S, Narasimha S, Ashok A, Dye C. An Investigation of the Information Sought by Caregivers of Alzheimer’s Patients on Online Peer Support Groups. Cyberpsychol Behav Soc Netw 2017;20(10):640-657. PMID:29039697

56. Schinköthe D, Wilz G. The Assessment of Treatment Integrity in a Cognitive Behavioral Telephone Intervention Study With Dementia Caregivers. Clin Gerontol 2014;37(3):211-234. doi:10.1080/07317115.2014.886653

57. Schinköthe D, Altmann U, Wilz G. The effects of treatment adherence and treatment-specific therapeutic competencies on outcome and goal attainment in telephone-based therapy with caregivers of people with dementia. Aging Ment Health 2015;19(9):808-817. PMID:25358445

58. Steffen AM, Gant JR. A telehealth behavioral coaching intervention for neurocognitive disorder family carers. Int J Geriatr Psychiatry 2016;31(2):195-203. PMID:26077904

59. Torkamani M, McDonald L, Saez Aguayo I, Kanios C, Katsanou M-N, Madeley L, Limousin PD, Lees AJ, Haritou M, Jahanshahi M. A randomized controlled pilot study to evaluate a technology platform for the assisted living of people with dementia and their carers. J Alzheimers Dis 2014;41(2):515-523. PMID:24643137

60. Tremont G, Davis JD, Papandonatos GD, Ott BR, Fortinsky RH, Gozalo P, Yue MS, Bryant K, Grover C, Bishop DS. Psychosocial telephone intervention for dementia caregivers: A randomized, controlled trial. Alzheimers Dement 2015;11(5):541-548. PMID:25074341

61. Tremont G, Davis JD, Ott BR, Galioto R, Crook C, Papandonatos GD, Fortinsky RH, Gozalo P, Bishop DS. Randomized Trial of the Family Intervention: Telephone Tracking-Caregiver for Dementia Caregivers: Use of Community and Healthcare Resources. J Am Geriatr Soc 2017;65(5):924-930. PMID:28008609

62. van Mierlo LD, Meiland FJM, van de Ven PM, van Hout HPJ, Dröes R-M. Evaluation of DEM-DISC, customized e-advice on health and social support services for informal carers and case managers of people with dementia; a cluster randomized trial. Int Psychogeriatr 2015;27(8):1365-1378. PMID:25872457

63. Williams K, Arthur A, Niedens M, Moushey L, Hutfles L. In-home monitoring support for dementia caregivers: a feasibility study. Clin Nurs Res 2013;22(2):139-150. PMID:22997349

64. Wilz G, Soellner R. Evaluation of a Short-Term Telephone-Based Cognitive Behavioral Intervention for Dementia Family Caregivers. Clin Gerontol 2016;39(1):25-47. doi:10.1080/07317115.2015.1101631

65. Wilz G, Meichsner F, Soellner R. Are psychotherapeutic effects on family caregivers of people with dementia sustainable? Two-year long-term effects of a telephone-based cognitive behavioral intervention. Aging Ment Health 2017;21(7):774-781. PMID:26954588

66. Wilz G, Reder M, Meichsner F, Soellner R. The Tele.TAnDem Intervention: Telephone-based CBT for Family Caregivers of People With Dementia. Gerontologist 2018;58(2):e118-e129. PMID:29190357

67. Wilz G, Weise L, Reiter C, Reder M, Machmer A, Soellner R. Intervention Helps Family Caregivers of People With Dementia Attain Own Therapy Goals. Am J Alzheimers Dis Other Deme 2018;33(5):301-308. PMID:29660988
