# Supplementary material for: Technology-Assisted Home Care for People With Dementia and Their Relatives: Scoping Review
Source: JMIR Aging. 2021 Jan 20;4(1):e25307. doi: 10.2196/25307 (PMC7857954; doi:10.2196/25307)
Supplement: Multimedia Appendix 4 [file aging_v4i1e25307_app4.docx]

**Appendix 4: Study details – both target groups**

| **Author** | **Study design** | **Study aim** | **Country**  **Setting** | **Number of participants** | **Technology name** | **Technology group** | **Technology aim** |
| --- | --- | --- | --- | --- | --- | --- | --- |
| Alwin et al. 2013 [1] | Repeated measures study | Exploration and evaluation | Sweden  home | 47 | Unclear | Various | Various |
| Boman et al. 2014 [2] | Case study | Usability | Sweden  home (lab) | 8 | Unclear | Videophone | Improvement of communication |
| Cavallo et al. 2015 [3] | Case study | Effectiveness and acceptability | Italy  home | 14 | Unclear | AAL  monitoring system | Improvement of safety, supporting communication and enabling cognitive stimulation |
| Dal Bello-Haas et al. 2014 [4] | Cross sectional study | Feasibility and acceptability | Canada  home and regional health authority | 77 | Unclear | Telehealth | Improvement of symptoms and skills (mobility) |
|  | Case study |  |  | 4 |  |  |  |
| Davison et al. 2016 [5] | RCT | Effectiveness, acceptability and barriers to use | Australia  nursing home | 16 | Memory Box | Personal computer | Improvement of symptoms and enabling reminiscence therapy |
| Ekström et al. 2017 [6] | Case study | Exploration and usability | Sweden  home | 2 | GoTalk NOW | Tablet  app | Improvement of communication |
| Hattink et al. 2016a [7] | RCT | Effectiveness and  usability | Germany  home | 11 | Rosetta | AAL system | Supporting performing ADLs and improving safety |
|  | CT |  | Netherlands, Germany and Belgium  home | 31 |  |  |  |
| Hattink et al. 2016b [8] | Cross sectional study with  mixed methods | Usability, usefulness and feasibility | Netherlands  home | observations 10 | Digital Alzheimer Center | Online portal and app | Improvement of care |
|  |  |  |  | survey 287 |  |  |  |
|  |  |  |  | interviews 18 |  |  |  |
| Laird et al. 2018 [9] | Repeated measures study | Effectiveness | UK  home | 60 | InspireD | Tablet  app | Enabling reminiscence therapy |
| Lazar et al. 2015 [10] | Case study | Feasibility | USA  nursing home | 2 | Various apps | Touchscreen computer with various applications and input devices | Supporting social interaction, exercises, reminiscence therapy and cognitive stimulation |
| Lazar et al. 2016 [11] | Case study | Feasibility | USA  nursing home | 9 | Various apps: iN2L Mobile Lite Package, It`s never 2 Late, Centennial, CO | Touchscreen computer with various applications and input devices | Supporting social interaction, exercises, reminiscence therapy and cognitive stimulation |
| McKenzie et al. 2013 [12] | Case study | Evaluation and proof of concept | USA  home | 12 | Various | Various | Improving of care and safety and supporting caregivers |
| Megges et al. 2017 [13] | Pre-post study | Usability and evaluation | Germany  home | 32 | webXells | Monitoring  GPS  app | Enabling monitoring |
| Moyle et al. 2014 [14] | Case study | Feasibility | Australia  nursing home | 11 | Giraff | Telepresence robot | Improvement of communication and social interaction |
| Navarro et al. 2014 [15] | Case study | Evaluation and usability | Mexico  home | 2 | AnswerBoard  AnswerPad | ambient assisted intervention system (AAIS) | Improvement of symptoms and engagement |
| Navarro et al. 2016 [16] | Case study | Effectiveness and adoption in occupational therapy | Mexico  home | 4 | AnswerBoard  AnswerPad | ambient assisted intervention system (AAIS) | Improvement of symptoms and engagement |
| Nijhof et al. 2013a [17] | Case series | Evaluation | Netherlands  home | 32 | Pal4-dementia system | Tablet  app | Enabling the PwD to continue living at home |
| Nijhof et al. 2013b [18] | Case series | Evaluation | Netherlands  home | 28 | ADLife system | Monitoring  sensor | Enabling monitoring |
| Olsson et al. 2013 [19] | Qualitative  study | Exploration | Sweden  home | 10 | Unclear | Monitoring  GPS | Enabling monitoring |
| Olsson et al. 2015 [20] | Case study | Effectiveness | Sweden  home | 6 | Unclear | Monitoring  GPS | Enabling monitoring |
| Purves et al. 2015 [21] | Qualitative study  (exclusion: no dementia) | Pilot testing | Canada  nursing home | 6 | Computer Interactive Reminiscence Conversation Aid (CIRCA) | Computer  touch screen  program | Improvement of communication and enabling reminiscence therapy |
|  | Case study |  |  |  |  |  |  |
| Robinson et al. 2013 [22] | Qualitative study | Exploration of suitability | New Zealand  nursing home | 21 | Guide and Paro | Robot | Various |
| Rodriquez 2013 [23] | Qualitative study | Exploration | Unclear | 32 (354 posts) | Unclear | Online forum | Enabling communication |
| Rostill et al. 2018 [24] | RCT | Effectiveness | UK  home | 408 | technology integrated health management (TIHM) | Monitoring  sensor | Enabling monitoring |
| Shaw et al. 2017 [25] | Case Study | Exploration of feasibility and usefulness | UK  home | 1 household | geo-fence | Monitoring  GPS | Enabling monitoring |
| Subramaniam et al. 2016 [26] | Case study | Evaluation | UK  nursing home | 12 | Unclear | TV video  digital life storybooks | Enabling reminiscence therapy |
| Teunissen et al. 2017 [27] | Qualitative study | Exploration | Netherlands  nursing home | Unclear | Interactice instrument CRDL | Interactice instrument  human artwork reponses | Improvement of communication |
| White et al. 2014 [28] | Qualitative study | Exploration | Unclear  home | 10 | Unclear | Monitoring  electronic tracking (GPS and sensor) | Enabling monitoring |

References

1. Alwin J, Persson J, Krevers B. Perception and significance of an assistive technology intervention - the perspectives of relatives of persons with dementia. Disabil Rehabil 2013;35(18):1519-1526. PMID:23311670

2. Boman I-L, Lundberg S, Starkhammar S, Nygård L. Exploring the usability of a videophone mock-up for persons with dementia and their significant others. BMC Geriatr 2014;14:49. PMID:24739662

3. Cavallo F, Aquilano M, Arvati M. An Ambient Assisted Living Approach in Designing Domiciliary Services Combined With Innovative Technologies for Patients With Alzheimer’s Disease: A Case Study. Am J Alzheimers Dis Other Deme 2015;30(1):69-77. PMID:24951634

4. Dal Bello-Haas VPM, O’Connell ME, Morgan DG, Crossley M. Lessons learned: feasibility and acceptability of a telehealth-delivered exercise intervention for rural-dwelling individuals with dementia and their caregivers. Rural Remote Health 2014;14(3):2715. PMID:25081991

5. Davison TE, Nayer K, Coxon S, Bono A, Eppingstall B, Jeon Y-H, van der Ploeg, Eva S., O’Connor DW. A personalized multimedia device to treat agitated behavior and improve mood in people with dementia: A pilot study. Geriatr Nurs 2016;37(1):25-29. PMID:26412509

6. Ekström A, Ferm U, Samuelsson C. Digital communication support and Alzheimer’s disease. Dementia (London) 2017;16(6):711-731. PMID:26643684

7. Hattink BJJ, Meiland FJM, Overmars-Marx T, Boer M de, Ebben PWG, van Blanken M, Verhaeghe S, Stalpers-Croeze I, Jedlitschka A, Flick SE, v/d Leeuw J, Karkowski I, Dröes RM. The electronic, personalizable Rosetta system for dementia care: exploring the user-friendliness, usefulness and impact. Disabil Rehabil Assist Technol 2016;11(1):61-71. PMID:24989993

8. Hattink B, Droes R-M, Sikkes S, Oostra E, Lemstra AW. Evaluation of the Digital Alzheimer Center: Testing Usability and Usefulness of an Online Portal for Patients with Dementia and Their Carers. JMIR Res Protoc 2016;5(3):e144. PMID:27444209

9. Laird EA, Ryan A, McCauley C, Bond RB, Mulvenna MD, Curran KJ, Bunting B, Ferry F, Gibson A. Using Mobile Technology to Provide Personalized Reminiscence for People Living With Dementia and Their Carers: Appraisal of Outcomes From a Quasi-Experimental Study. JMIR Ment Health 2018;5(3):e57. PMID:30206053

10. Lazar A, Demiris G, Thompson HJ. Involving family members in the implementation and evaluation of technologies for dementia: a dyad case study. J Gerontol Nurs 2015;41(4):21-26. PMID:25800405

11. Lazar A, Demiris G, Thompson HJ. Evaluation of a multifunctional technology system in a memory care unit: Opportunities for innovation in dementia care. Inform Health Soc Care 2016;41(4):373-386. PMID:26819070

12. McKenzie B, Bowen ME, Keys K, Bulat T. Safe home program: a suite of technologies to support extended home care of persons with dementia. Am J Alzheimers Dis Other Deme 2013;28(4):348-354. PMID:23677733

13. Megges H, Freiesleben SD, Jankowski N, Haas B, Peters O. Technology for home dementia care: A prototype locating system put to the test. Alzheimers Dement (N Y) 2017;3(3):332-338. PMID:29067340

14. Moyle W, Jones C, Cooke M, O’Dwyer S, Sung B, Drummond S. Connecting the person with dementia and family: a feasibility study of a telepresence robot. BMC Geriatr 2014;14:7. PMID:24456417

15. Navarro RF, Rodríguez MD, Favela J. Intervention tailoring in augmented cognition systems for elders with dementia. IEEE J Biomed Health Inform 2014;18(1):361-367. PMID:24403435

16. Navarro RF, Rodriguez MD, Favela J. Use and Adoption of an Assisted Cognition System to Support Therapies for People with Dementia. Comput Math Methods Med 2016;16:1075191. PMID:27648106

17. Nijhof N, van Gemert-Pijnen J, Burns CM, Seydel ER. A personal assistant for dementia to stay at home safe at reduced cost. Gerontechnology 2013;11(3):469-479. doi:10.4017/gt.2013.11.3.005.00

18. Nijhof N, van Gemert-Pijnen LJEWC, Woolrych R, Sixsmith A. An evaluation of preventive sensor technology for dementia care. J Telemed Telecare 2013;19(2):95-100. PMID:23434539

19. Olsson A, Engström M, Lampic C, Skovdahl K. A passive positioning alarm used by persons with dementia and their spouses—a qualitative intervention study. BMC Geriatr 2013;13:11. PMID:23384329

20. Olsson A, Engström M, Åsenlöf P, Skovdahl K, Lampic C. Effects of tracking technology on daily life of persons with dementia: three experimental single-case studies. Am J Alzheimers Dis Other Deme 2015;30(1):29-40. PMID:24771764

21. Purves BA, Phinney A, Hulko W, Puurveen G, Astell AJ. Developing CIRCA-BC and exploring the role of the computer as a third participant in conversation. Am J Alzheimers Dis Other Deme 2015;30(1):101-107. PMID:24928817

22. Robinson H, MacDonald BA, Kerse N, Broadbent E. Suitability of healthcare robots for a dementia unit and suggested improvements. J Am Med Dir Assoc 2013;14(1):34-40. PMID:23098418

23. Rodriquez J. Narrating dementia: self and community in an online forum. Qual Health Res 2013;23(9):1215-1227. PMID:23907588

24. Rostill H, Nilforooshan R, Morgan A, Barnaghi P, Ream E, Chrysanthaki T. Technology integrated health management for dementia. Br J Community Nurs 2018;23(10):502-508. PMID:30290728

25. Shaw J, Shaw S, Wherton J, Hughes G, Greenhalgh T. Studying Scale-Up and Spread as Social Practice: Theoretical Introduction and Empirical Case Study. J Med Internet Res 2017;19(7):e244. PMID:28687532

26. Subramaniam P, Woods B. Digital life storybooks for people with dementia living in care homes: an evaluation. Clin Interv Aging 2016;11:1263-1276. PMID:27698556

27. Teunissen L, Luyten T, Witte L de. Reconnecting People with Dementia by Using the Interactive Instrument CRDL. Stud Health Technol Inform 2017;242:9-15. PMID:28873769

28. White EB, Montgomery P. Electronic tracking for people with dementia: an exploratory study of the ethical issues experienced by carers in making decisions about usage. Dementia (London) 2014;13(2):216-232. PMID:24599815
